# Design and Synthesis of New 5-Methylisatin Derivatives as Potential CDK2 Inhibitors

**DOI:** 10.3390/ijms26052144

**Published:** 2025-02-27

**Authors:** Przemysław Czeleń, Agnieszka Skotnicka, Beata Szefler, Janina Kabatc-Borcz, Paweł Sutkowy

**Affiliations:** 1 Department of Physical Chemistry, Faculty of Pharmacy, Collegium Medicum, Nicolaus Copernicus University, Kurpinskiego 5, 85-096 Bydgoszcz, Poland; beatas@cm.umk.pl; 2 Faculty of Chemical Technology and Engineering, Bydgoszcz University of Science and Technology, Seminaryjna 3, 85-326 Bydgoszcz, Poland; askot@pbs.edu.pl (A.S.); nina@pbs.edu.pl (J.K.-B.); 3 Department of Medical Biology and Biochemistry, Faculty of Medicine, Collegium Medicum, Nicolaus Copernicus University, Karłowicza 24, 85-092 Bydgoszcz, Poland; p.sutkowy@cm.umk.pl

**Keywords:** 5-methylisatin, CDK2, competitive inhibition, molecular dynamics, synthesis, spectroscopic properties

## Abstract

Cancer remains one of the leading causes of death globally, driving the need for effective therapies. Targeting cyclin-dependent kinase 2 (CDK2), a critical cell cycle regulator, is a promising approach for cancer treatment. This study developed a new group of 5-methylisatin derivatives with strong binding potential to CDK2. By combining the isatin core with various benzoylhydrazide substituents, the design process was guided by molecular docking, dynamic simulations, and ADMET analysis. Thirty-one derivatives were modelled, and a subset was synthesised and characterised for their physicochemical and spectroscopic properties. The analysis suggested that substitutions at R2 and R3 positions improved binding affinity, while modifications at R4 were less favourable. Hydrogen bonds with GLU81 and LEU83, along with hydrophobic interactions, were key to stabilising the complexes. A comparison with a reference molecule (**RM**) 3-((2,6-Dichlorobenzylidene)hydrazono)indolin-2-one, showing inhibitory activity similar to doxorubicin, revealed several advantages for the new derivatives. The multidimensional comparative analysis highlighted significant improvements in active site affinity, conformational stability, and fit. ADMET analysis confirmed comparable performance in most areas, with superior bioavailability observed in derivatives **1**, **2a**, **2b**, **3h**, **3b**, and **3e**. These results suggest that 5-methylisatin derivatives could be promising CDK2 inhibitors.

## 1. Introduction

Malignant diseases remain one of the leading causes of mortality worldwide [1,2], highlighting the need for ongoing research into new and effective therapeutic agents. Among the various strategies used to address this condition, targeting enzymes that regulate cellular processes offers a promising approach. Cyclin-dependent kinases (CDKs), particularly CDK2, play a crucial role in cell cycle regulation, transcription, and replication [3,4,5]. Overexpression of CDK2 is often associated with uncontrolled cell proliferation, a hallmark of cancer, making the inhibition of CDK2 activity a valuable therapeutic strategy [6,7,8,9,10]. The key interaction site for potential inhibitors within the active site is the DFG motif, comprising Asp145-Phe146-Gly147, along with Leu83 and Asp86, which directly participate in ATP interactions through a network of hydrogen bonds. Additionally, Glu81 and Leu83, which form the hinge linker sequence responsible for kinase flexibility, play a crucial role in protein activity [11]. The adenine pocket, where GLU81 and LEU83 play a key role, is a critical target in the strategy for inhibiting the CDK2 active site. Numerous classes of chemical compounds have been developed, designed with specific structural features that incorporate a hydrogen bond donor–acceptor–donor sequence. Notable examples include NU6102 [12,13], (R)-roscovitine [14,15], Tri-cycle (Milciclib) [16,17], 2-anilinopyrimidine derivatives [18,19], as well as their related analogues. The significant structural similarities among enzymes classified as cyclin-dependent kinases make the presented groups of compounds likely to exhibit substantial inhibitory activity against CDK2, CDK1, CDK5, CDK7, CDK8, CDK9, and CDK12. Isatin derivatives containing an oxindole core in their structure represent another group of compounds with significant inhibitory potential against CDK2 [7,20,21,22]. This scaffold is known for its ability to interact with the active site of CDK2, preventing the enzyme from facilitating cell cycle progression. Previous studies have demonstrated that isatin-based compounds can effectively bind to and inhibit CDK2, making them suitable candidates for anticancer drug development. The structural similarity between the active sites of CDK2 and other kinases [23,24,25] suggests that isatin derivatives may have broad-spectrum inhibitory effects [20,26,27,28,29,30,31,32,33]. The development of CDK inhibitors, including those derived from isatin, has been extensively documented [7,34]. Certain isatin derivatives exhibit potent inhibitory activity against CDK2 and related kinases, inducing cell cycle arrest and apoptosis in various cancer cell lines. Computational chemistry methods, such as molecular docking and dynamics simulations, have been instrumental in designing isatin derivatives with enhanced binding affinity and selectivity for CDK2 [35,36,37]. These methods allow for precise modelling of inhibitor–enzyme interactions, providing insights into the structural and energetic factors contributing to effective inhibition. The design of isatin derivatives as CDK2 inhibitors involves strategic modifications to the oxindole core to enhance binding affinity and selectivity. By incorporating various substituents at specific positions on the isatin molecule, researchers aim to optimise interactions with key amino acids in the CDK2 active site. Previous studies have shown that derivatives based on 5-nitroisatin and substituted benzoylhydrazines can be synthesised to create a group of compounds exhibiting significant inhibitory capabilities towards the active site of CDK2 [35,38]. Based on these earlier results, it seems both interesting and justified to investigate the properties of other modifications to the isatin core as a foundation for developing new potential anticancer drugs. Molecular docking studies guide these modifications, highlighting the importance of hydrogen bonding, hydrophobic interactions, and other non-covalent forces in stabilising the inhibitor–enzyme complex. Isatin derivatives represent a promising class of CDK2 inhibitors with potential applications in cancer therapy. Their development could provide a valuable addition to the arsenal of anticancer therapies, offering new hope in the fight against this devastating disease.

## 2. Results and Discussion

### 2.1. Design and Computational Analysis of Binding Activity

**Figure 1:**
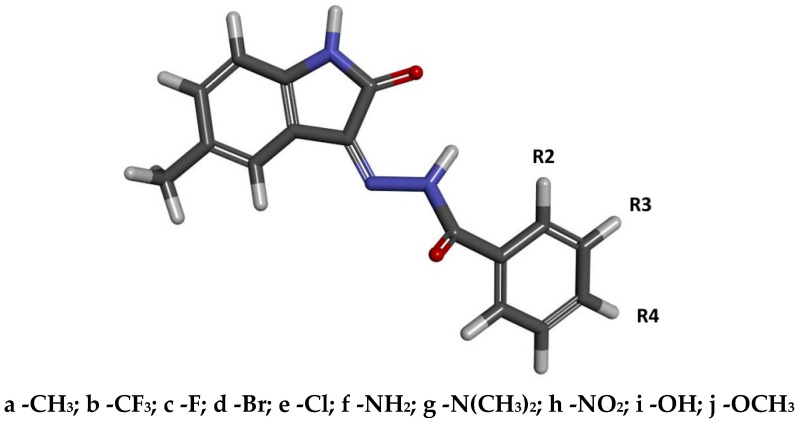
The graphical representation of 5-methylisatin-based benzoylhydrazines. The markings R2, R3, and R4 represent the places of substitution of chemical groups. The designations of individual derivatives were generated based on the number defining the substituent’s location in the aromatic ring and the letter assigned to the functional group used.

The design of the compounds considered in this study involved utilising the molecular core of substituted isatin and combining it with an additional substituent that could significantly increase its affinity for the enzyme’s active site. Previous work clearly indicated that modified isatin derivatives serve as a good platform for creating new substances with pharmacological potential in the context of cyclin-dependent kinase (CDK) inhibition. The conducted studies demonstrated that isatin derivatives substituted at the fifth position with a methyl group exhibit biological activity very similar to that of 5-nitroisatin derivatives studied in the previous work [35]. The compounds considered here are the products of the reaction between 5-methylisatin and substituted benzoylhydrazide derivatives. During the design stage, benzoylhydrazide derivatives with functional groups capable of different types of interactions—such as hydrogen bonding, halogen interactions, and hydrophobic contacts—with amino acids in the active site were considered. The model structure of the studied compounds is presented in Figure 1, where the labels R2, R3, and R4 indicate the locations of substituents in the aromatic system.

**Figure 2:**
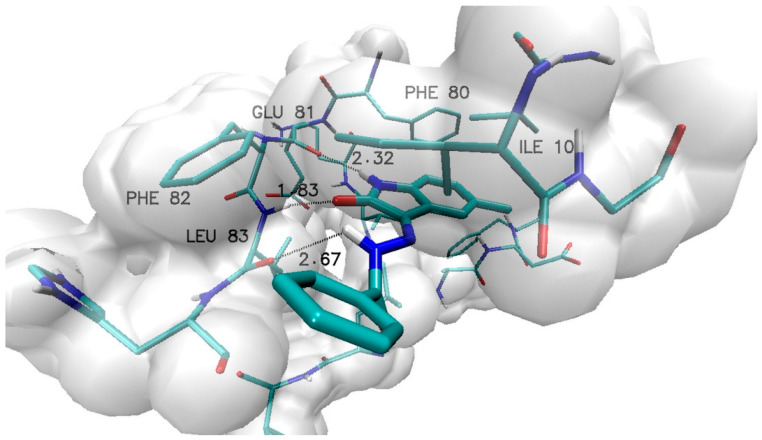
Graphic representation of *N*′-[5-methyl-2-oxo-1,2-dihydro-3*H*-indol-3-ylidene]benzohydrazide (1) complex with active site of CDK2 enzyme.

Based on the selected group of substituents, thirty-one derivatives of 5-methylisatin were created, taking into account both the native and modified forms of benzoylhydrazide. The docking procedures conducted for the active site of cyclin-dependent kinase 2 (CDK2) allowed for the formation of complexes for each of the proposed derivatives. The graphical representation of the complex formed by the unsubstituted derivative of 5-methylisatin is shown in Figure 2. The inhibitor molecule is tightly positioned in the hydrophobic pocket of the active site, where the side chains of phenylalanines PHE 80 and PHE 82, as well as isoleucine ILE 10, play a significant role. The inhibitor molecule is also involved in forming hydrogen bonds with glutamic acid GLU 81 and leucine LEU 83. The observed interactions are typical for most of the obtained complexes.

The results describing the chemical affinity and inhibition constant for each inhibitor relative to the active site of the enzyme are presented in Table 1. The values of the inhibition constant were determined based on the Van’t Hoff equation.

$$IC={exp}^{\left(\frac{{\Delta G}_{b}}{RT}\right)}$$

The presented summary of the discussed parameters clearly indicates that modifications at positions R2, R3, and R4 affect ligand binding abilities in different ways and are strictly dependent on the type of substituent used.

For substitutions at position R2, the greatest variability in affinity values is observed (1.5 kcal/mol), with the highest values recorded for groups that serve as hydrogen bond donors or can participate in halogen interactions. Derivatives with bulky chemical groups at position R2, which may cause steric hindrance (**2h**, **2g**, **2f**), exhibited significantly lower binding abilities. Very similar relationships can be observed with substitutions at position R3; however, the discrepancies in binding abilities among the analysed derivatives were significantly smaller (0.74 kcal/mol) compared to the previously analysed group of compounds. The most homogeneous group of derivatives in terms of binding abilities were ligands with substitutions at position R4. Excluding the **4b** derivative from the analysis, the range of affinity values for all remaining derivatives would be only 0.34 kcal/mol. This clearly indicates that modifications at this position are the least significant for differentiating the binding abilities of the designed derivatives in the context of interaction with the CDK2 active site. The use of the three fluoromethyl group for each type of substitution caused the greatest increase in the binding abilities of potential CDK2 inhibitors, as evidenced by the affinity values and inhibition constants obtained for derivatives **2b**, **3b**, and **4b**. A significant increase in binding abilities is also observed in the case of molecules where an amino group is used at positions R2 and R3 (**2f**, **3f**). Similar binding abilities towards the active site were also noted for derivatives containing halogen substituents, as well as methyl and nitro groups (**2a**, **2d**, **3e**, **3h**). For most complexes obtained during the docking stage, similar conformational properties of inhibitors within the active site are observed. Table 2 presents the lengths of the most significant hydrogen bonds and distances between selected hydrophobic centres. Their analysis shows that the modifications introduced in the inhibitors most significantly affect the displacement of hydrophobic centres, and the length of the hydrogen bond formed with the oxygen atom of LEU83. The least variability was noted for the hydrogen bonds formed by the isatin core with GLU81 and LEU83. Considering the geometric criteria for classifying hydrogen bonds, both can be classified as medium-strength interactions, whereas the bond formed by the hydrogen atom from the hydrazine group with Leu 83 O should be classified as a weak bond.

To provide a fully accurate evaluation of the obtained results, it is important to compare them with the characteristics of compounds of similar structure that have demonstrated inhibitory capabilities against the biological target in question. Due to its chemical similarity, the reference molecule (**RM**) 3-((2,6-Dichlorobenzylidene)hydrazono)indolin-2-one was selected. In vitro studies have shown that this molecule has a comparable inhibitory ability against the CDK2 active site to that of doxorubicin, a commonly used drug in cancer treatment [39].

**Figure 3:**
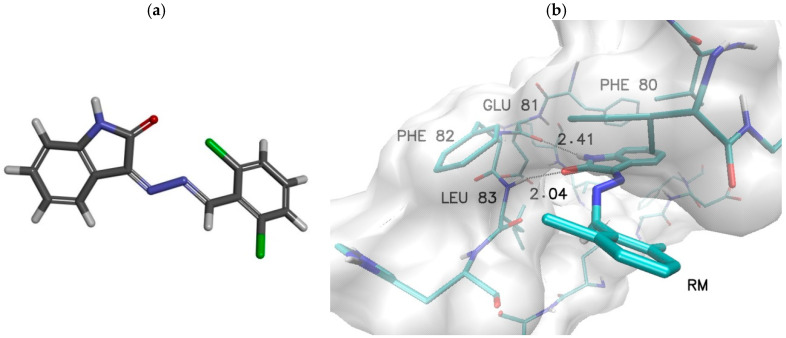
Graphical representation of reference molecule (**RM**) 3-((2,6-Dichlorobenzylidene)hydrazono)indolin-2-one (**a**). Graphical representation of **RM** complex with active site of CDK2 enzyme (**b**) obtained during docking stage.

Table 2’s active sites are presented in Figure 3. It can be unequivocally stated that the isatin core of the selected reference compound plays a similar role in stabilising the inhibitor within the active site as in the case of the molecules studied in this work. This is demonstrated by the analogous orientation relative to the amino acids and the interactions identified during the docking stage. The affinity value obtained for the interaction between the reference molecule and the enzyme (−8.62 kcal/mol) is significantly lower than the values observed for the new isatin derivatives considered in this study. In the most extreme cases, the difference exceeds 1.2 kcal/mol. The structure of the complex formed by the reference molecule is similar to the complexes of the compounds under investigation; however, certain differences can be observed. The isatin core is similarly positioned in the hydrophobic pocket of the active site, as indicated by the distance between the aromatic systems of the core and phenylalanine PHE80. However, in the case of hydrogen bonds with GLU81 and LEU83, the distances are significantly greater, which may suggest a slightly weaker stabilising effect. The most pronounced differences between the studied inhibitors and the reference molecule are observed in the aromatic systems attached to the isatin core via a bonding system. The presence of two chlorine atoms in positions 2 and 6 hinders the adoption of conformations that would allow for stacking interactions between the aromatic systems of the inhibitor and PHE82, leading to a reorientation of the aromatic system outside the hydrophobic pocket. The comparison of the energetic and structural characteristics of the complexes formed by the newly designed 5-methylisatin derivatives with the reference system suggests that they may be more effective inhibitors of the CDK2 enzyme.

Based on the obtained chemical affinity values and the availability of benzoylhydrazide derivatives necessary for synthesis, a group of inhibitors was selected and introduced into the synthesis phase and more advanced computational analysis based on molecular dynamics simulations. The initial assessment of inhibitor complexes with the CDK2 protein referred to the dynamic conformational stability of the primary systems obtained at the docking stage. Molecular dynamics simulations allow for an in-depth evaluation of the structural properties of such systems and their evolution over time. The data presented in Table 3 and Figure S1 show the root mean square deviation (RMSD) values, which describe the structural variability of the analysed complexes relative to the initial system. They allow for visualisation of the dynamics and the scale of conformational changes in the analysed inhibitors within the active site of CDK2. In most simulations, we can observe that the structure of the active site undergoes significant relaxation during the first 20 ns of molecular dynamics, as evidenced by the increasing trend in RMSD values, which stabilise in the initial period of molecular dynamics (Figure S1). The conducted statistical testing clearly indicates that this effect concerns the structural properties of the protein to a much greater extent than the ligands themselves. For the enzyme, statistically significant changes in the RMSD data were observed in 10 out of the 11 populations studied, while for the ligands, only four showed such changes. These observations, however, suggest excluding the initial stage of the simulation from further analyses focused on conformational properties and binding affinity. The analysis of values describing CDK2 inhibitors indicates that they can be fundamentally divided into three groups. The first group consists of molecules that adopted an optimal conformation relative to the active site during the docking stage (**1**, **2b**, **2d**, **2f**, **3f**, **3h**) and exhibit minimal structural fluctuation during the simulation. The second includes molecules that attained a stable conformation through conformational changes during molecular dynamics (**2a**, **3b**). In both cases, the recorded conformational changes are associated with the rotation of the aromatic system located in the side chain; the representative geometries of such systems are presented in Figure S2. The final group comprises molecules that exhibit significant conformational variability throughout the simulation, indicating improper conformational fitting to the active site (**3e**, **4b**). The inability to attain a stable conformation that ensures a lasting binding with the active site indicates the presence of some form of steric hindrance, which prevents the formation of a stable complex with the CDK2 active site. The complex of the reference molecule with CDK2 exhibits considerable conformational stability. The RMSD values describing both the ligand and the active site indicate structural stabilisation of the system obtained during docking, with minimal conformational fluctuations. This is further confirmed by the low standard deviation values and the uniform distribution of data throughout the simulation.

**Figure 4:**
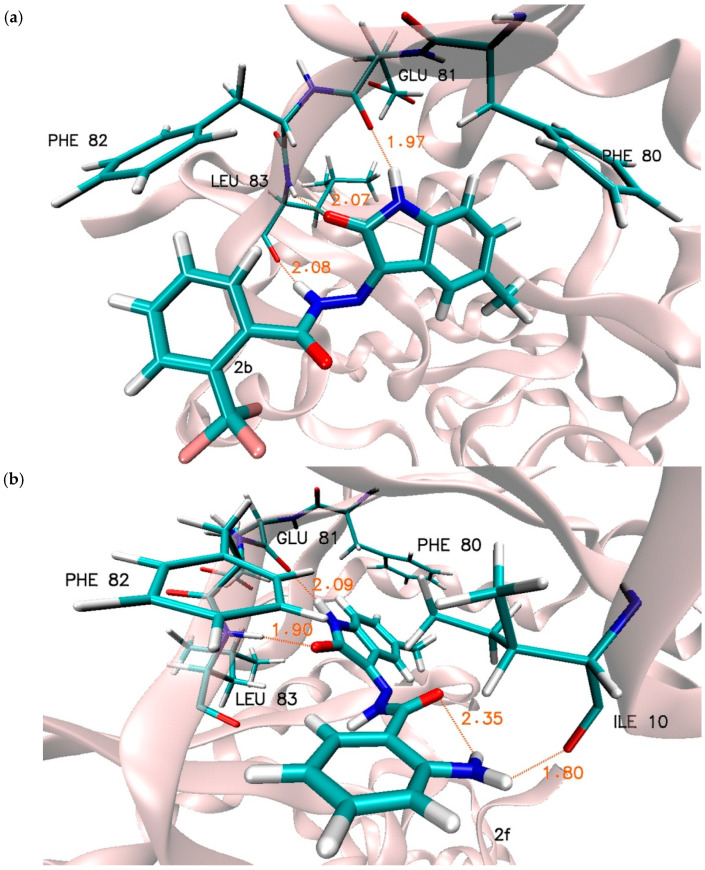

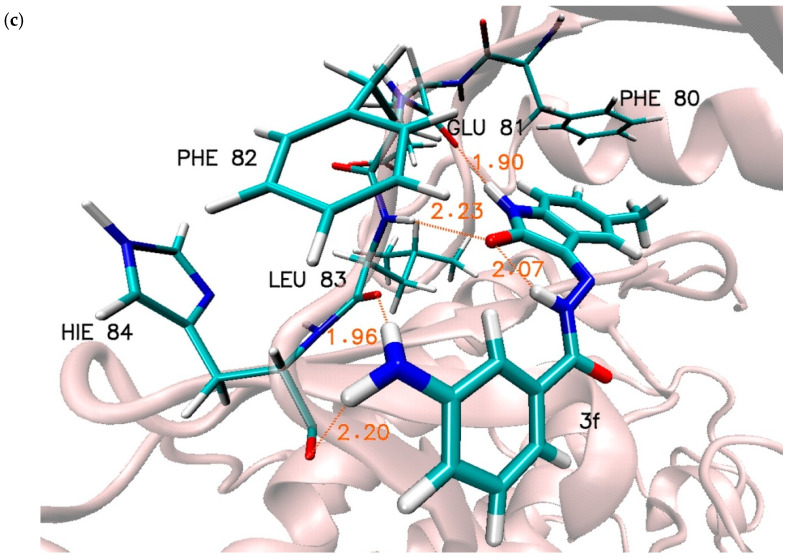
Graphic representation of interactions involved in stabilisation of CDK2 complex with chosen 5-methylisatin-based benzoylhydrazines, (**a**) with **2b**, (**b**) with **2f**, and (**c**) with **3f**. Presented systems correspond to most representative conformational arrangements.

The stability of the considered complexes is maintained through various types of interactions, including hydrogen bonds and hydrophobic interactions. In the preliminary analysis of the complexes obtained during the docking stage, three hydrogen bonds were identified in each of the analysed systems. These bonds involve oxygen and hydrogen atoms from the isatin core, as well as a hydrogen atom from the hydrazine group. The stability of these types of interactions, as well as the potential for other interactions involving functional groups located on the benzoyl hydrazine ring, were verified during molecular dynamics simulations. Table 4 presents a cumulative analysis of hydrogen bonds identified within the active site space for complexes formed by the selected inhibitor molecules. The applied analysis not only confirms the presence of a given type of interaction but also illustrates its quality and variability, taking into account the entire population of conformers obtained during the molecular dynamics simulations. Based on the attached data, we can unequivocally state that the hydrogen bonds formed with GLU81 and LEU83 by the atoms of the isatin core are extremely stable in all conformers gathered for each of the analysed complexes. Comparing the obtained distributions with the bond lengths identified for the complexes obtained during docking, a significant change characteristic for all complexes can be observed. Specifically, in the systems derived from molecular dynamics, there is a decrease in the bond length of Ligand (H1) … (O) GLU 81, accompanied by an increase in the number of conformers with a longer bond length of Ligand (O1) … (HN) LEU 83. This indicates a change in the orientation of the isatin core relative to the active site. We can unequivocally observe this in Figure 4, which depicts the selected ligand complexes obtained during the molecular dynamics simulations. Analysing all the obtained data populations, it can be stated that in the case of bonds formed with GLU81, at least 88% of the considered conformers form a medium-strength hydrogen bond, whereas for LEU83, this value drops to 75%. These values clearly indicate the significant role of these interactions in stabilising the complexes. The third of the interactions identified during docking, namely the hydrogen bond formed by the hydrazine hydrogen atom with the oxygen atom of LEU83, has a much more labile character. The location of the hydrogen bond donor in the side chain is a factor that significantly increases the possibility of rotation induced by various types of interactions involving the aromatic system associated with the core of the molecule. Among all the considered systems, this interaction is observed for almost all conformers only in the complex formed by the **3f** molecule. In at least 60% of these conformers, it meets the criteria for a medium-strength hydrogen bond. In the case of the remaining complexes, the significance of this bond essentially decreases. While it is noted for a substantial percentage of conformers (**2b**—55.5%, **3b**—46.6%, **3h**—44.1%), the geometric criteria clearly indicate a predominance of weak hydrogen bonds. In addition to the interactions identified during docking, new hydrogen bonds were also found that formed through conformational changes in the ligands and the surroundings created by the amino acids of the active site. In the complex formed by the **3f** molecule, two interesting interactions can be observed. The first is a hydrogen bond located between the amine group and the oxygen atom of histidine HIE 84. This bond is observed in 97% of the conformers gathered during molecular dynamics, with a significant proportion of these conformers meeting the criteria for medium-strength hydrogen bonds. An additional stabilising factor for this inhibitor molecule in the complex is an internal hydrogen bond located between the oxygen atom of the isatin core and the hydrogen atom of the hydrazine group (95% of conformers). New interactions were also identified in the case of the **2f** molecule, involving hydrogen atoms from the amine group located at the second position of the aromatic ring. The first interaction is a hydrogen bond formed with the oxygen atom of isoleucine ILE 10, observed in 62.4% of the gathered conformers, mainly noting weak hydrogen bonds. This interaction is further stabilised by an intramolecular hydrogen bond that restricts the rotation of the inhibitor’s side chain, involving hydrogen atoms from the amine group and the oxygen atom from the benzoylhydrazine group. Its presence was observed in 98% of the analysed structures. The chemical structure of the reference molecule allows the formation of two hydrogen bonds involving the hydrogen and oxygen atoms of the isatin core. Molecular dynamics analysis for the complex formed with CDK2 confirms the presence of two hydrogen bonds, specifically with GLU81 and LEU83, observed in nearly all conformers collected during the simulation. The values presented in Table 4 highlight that the distribution of distances, and thus the stabilising effect of these bonds, differs from those recorded for the 5-methylisatin derivatives. The most noticeable difference is found in the bond with LEU83, which, in most of the analysed conformers, exhibits characteristics of weak hydrogen bonds.

**Figure 5:**
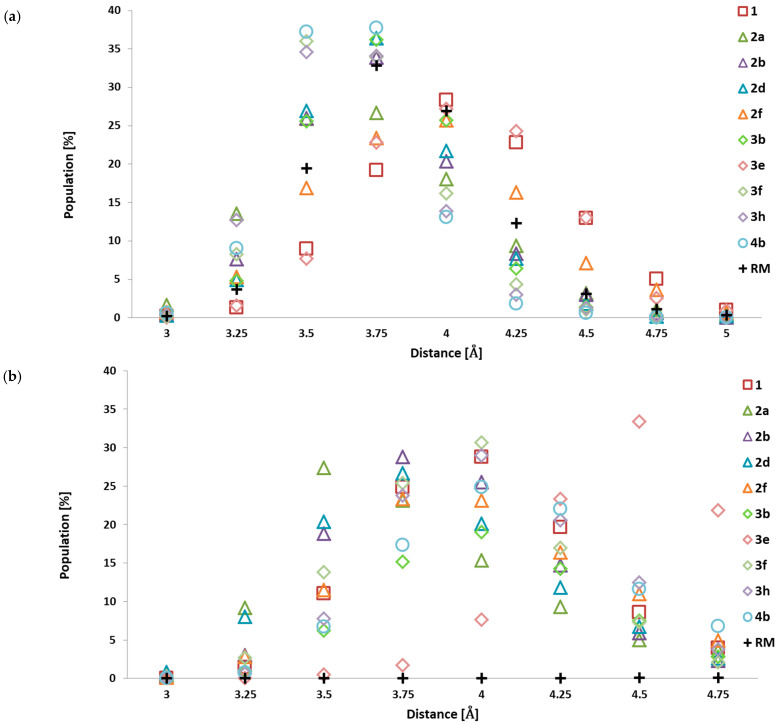
The cumulative analysis of the distances between aromatics systems of 5-methylisatin-based benzoylhydrazines and phenyloalanines PHE80 (**a**) and PHE 82 (**b**). The distances presented in chart labels represent middle values of intervals with a width of 0.5 Å.

An important aspect stabilising the ligand–protein complexes are hydrophobic interactions, one of which is a stacking interaction that can be attributed to interacting aromatic systems. In the structure of the analysed inhibitors, two aromatic systems can be observed that play a key role in positioning the inhibitor in the hydrophobic pocket, which also contains aromatic systems belonging to phenylalanines PHE 80 and PHE 82. Their location within the active site predisposes them to form stacking interactions with the considered 5-methylisatin derivatives, as can be observed in Figure 2 and Figure 3. To illustrate changes in the mutual arrangement of aromatic systems belonging to the ligands and the active site, the change in their mutual distance was analysed based on the distance between carbon atoms from the aromatic systems. The cumulative analysis, covering the data populations gathered for all conformers obtained during the molecular dynamics simulations, is presented in Figure 5. The presented data clearly indicate that for most modified derivatives of 5-methylisatin, significant changes are observed compared to the molecule without any substituents in the aromatic ring. The change in the orientation of the ligands relative to the hydrophobic pocket affects the entire molecule, not just the modified fragment. The analysis of conformational changes in the isatin core relative to the aromatic system of phenylalanine PHE 80 indicates that modifications in the inhibitors cause a significant decrease in the distance between these systems, by approximately 0.5 Å, compared to the native molecule, where the dominant distance oscillates around 4 Å. The greatest discrepancies can be observed in the case of the **4b**, **3f**, and **3h** derivatives. Among all the developed derivatives, only the **3e** molecule does not show significant changes in the orientation of the isatin core relative to the active site; the distributions obtained for it overlap with the native system. For the second aromatic system present in the analysed inhibitors, there is a greater variation in the distance relative to the aromatic ring of phenylalanine PHE 82. A group of derivatives can be distinguished in which there is a significant reduction in the distance between the aromatic systems, noticeable in the case of molecules **2a**, **2b**, and **2d** (dominant distance ~3.5 Å). For the remaining derivatives, the distance distribution indicates that the majority of the population is in the range of 3.75 to 4 Å. An exception is the **3e** molecule, for which a significant increase in the distance between the aromatic systems (~4.5 Å) is observed, indicating a greater protrusion of this molecule from the hydrophobic pocket. The increased conformational diversity observed for these fragments of the analysed derivatives results from the presence of relatively large functional groups in the aromatic system and their adjustment to the dynamically changing structure of the active site during molecular dynamics simulations. The conformational properties of the aromatic systems of the reference molecule differ significantly from those observed in the studied group of 5-methylisatin derivatives. When analysing the localisation of the isatin core and its mobility within the active site, it is evident that seven of the ten newly proposed inhibitors are more tightly embedded in the hydrophobic pocket of the active site and exhibit significantly lower mobility than the corresponding fragment of the reference molecule. A completely different characteristic is attributed to the additional aromatic system located in the side chain. The conducted studies clearly demonstrate that this system does not participate in interactions with the hydrophobic pocket, which is crucial for creating interactions with the corresponding part in the newly proposed group of compounds.

**Figure 6:**
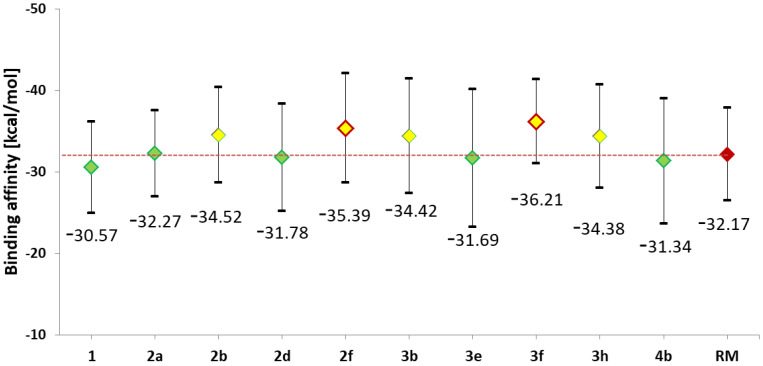
The values of enthalpic contribution to the binding affinity [kcal/mol] estimated for all complexes considered during the molecular dynamics stage. For values marked with yellow indicators, statistical analysis based on one-way analysis of variance (ANOVA) with the pairwise multiple comparison Holm–Sidak method showed a statistically significant difference compared to values obtained for the native system (1). For markers within the red frame, a significant difference was observed relative to the reference molecule (**RM**).

The structural analysis of complexes obtained during molecular dynamics simulations was extended by energy analysis using the molecular mechanics Poisson–Boltzmann surface area (MMPBSA) method. The calculations included randomly selected conformers from the last 60 ns of molecular dynamics, for which the enthalpic contributions to the binding affinity of the studied complexes were determined. A summary of the obtained values is presented in Figure 6. The values presented on the chart correspond to the average affinity values for each ligand, while the graphical markers represent the spread of values determined based on the standard deviation. For all derivatives, an increase in binding ability relative to the native molecule is observed; however, considering the RMSD values, it should be noted that for complexes **4b** and **3e**, significantly less favourable conformations in terms of energy were observed compared to the native molecule. The conducted statistical analysis clearly shows that for derivatives such as **3f**, **2f**, **2b**, **3b**, and **3h**, a statistically significant increase in binding affinity is observed compared to the values characterising the native system. The enthalpic contributions to the binding affinity determined for the reference molecule place its inhibitory abilities against the CDK2 active site within the range of values obtained for the studied group of 5-methylisatin derivatives. The spread in affinity values observed for the population of reference molecule conformers indicates the structural and energetic stability of the investigated complexes. The comparison of affinities between the new inhibitor candidates and the reference molecule suggests that within the selected group of 5-methylisatin derivatives, there are molecules with the potential to exhibit promising activity towards the CDK2 active site, especially **2f** and **3f**.

### 2.2. The Analysis of ADMET Properties

An important aspect of drug design is the evaluation of their properties in the context of molecular characteristics, absorption, metabolism, distribution, and toxicity. In this regard, we can rely on a wide range of methods based on quantitative structure–activity relationship (QSAR) analysis. The use of tools enabling comprehensive ADMET (Absorption, Distribution, Metabolism, Excretion and Toxicity) analysis significantly enhances the evaluation of the pharmacological suitability of newly developed drugs [40,41,42,43]. The appropriate analysis was conducted for all synthesised 5-methylisatin derivatives as well as the reference molecule. A set of descriptors was selected for evaluating the studied molecules, allowing for a comprehensive analysis of various aspects of their properties. The collected data are presented in Table 5. The first value presented in the table is molecular weight (MW), which for the considered molecules ranges from 279.1 to 357.07 u. All the molecules have molecular weights significantly below the threshold value (MW < 500 u) [44] that could indicate potential absorption issues via the oral route. For four of the proposed derivatives, the values are lower than those obtained for the reference molecule.

The next two columns display the number of hydrogen bond donors (nHD) and hydrogen bond acceptors (nHA), respectively. In both cases, all proposed 5-methylisatin derivatives meet the threshold requirements for compounds that can be used as drugs [44]. All the designed derivatives show a higher number of hydrogen bond donors and acceptors compared to the reference molecule; however, the discrepancies are slight and mostly related to a single unit increase.

The next analysed value, namely the Topological Polar Surface Area (TPSA), is an indicator closely associated with the presence of polar groups and is a key determinant of the absorption and distribution abilities of the studied substances. All the proposed compounds exhibit TPSA values significantly below 140 [45], which if exceeded could indicate potential absorption issues. Comparing their values to the reference molecule, it can be observed that the presence of a greater number of hydrogen bond donors and acceptors has also resulted in higher values of this parameter. The slightly higher TPSA values of the proposed derivatives are likely to result in a somewhat lower ability of the studied compounds to penetrate areas where increased lipid solubility is crucial, such as the central nervous system. However, this may lead to proportionally greater activity of these substances in peripheral actions. Log*P*, the octanol/water partition coefficient, LogD, and water solubility are additional parameters used to assess the bioavailability of the analysed chemical compounds. The values presented in Table 5 clearly indicate that for all the considered 5-methylisatin derivatives, the Log*P* values range from 2.711 to 3.848, which places them within the acceptable range for potential drugs. These values are significantly better than those of the reference molecule (4.462). The observed range for LogD values is 2.153 to 3.111, with only the **3b** and **3h** derivatives falling outside the optimal range. The estimated solubility values for the studied compounds range from −4.584 to −5.512, which are markedly better than the value for the reference molecule (−6.693). Low water solubility poses a challenge for drug bioavailability; however, studies have shown that the use of medical solvents or binary solvents based on water with slight additions of organic solvents can significantly help address this issue [38]. The descriptor values discussed so far form the basis for creating rules to evaluate the drug-likeness of new compounds with pharmacological potential. One of the most widely used is Lipinski’s Rule of Five, which is based on molecular descriptors such as MW (≤500), log*P* (≤5), nHA (≤10), and nHD (≤5) [44]. The values discussed so far clearly indicate that each of the proposed 5-methylisatin derivatives meets Lipinski’s Rule. The BBB (blood–brain barrier) values collected for the studied group of compounds indicate that most of the proposed derivatives should exhibit reduced permeability between the blood and the central nervous system. However, some uncertainty arises with derivatives **2f** and **3f**, for which, similarly to the reference molecule, there is an estimated ~80% probability of good ability to cross the BBB. Madin−Darby Canine Kidney (MDCK) is a model developed for screening membrane permeability, often correlated with BBB values. The collected data indicate that, for most of the studied molecules, including the reference molecule, high passive permeability is observed. Only in the case of derivatives **2f** and **3f**, permeability abilities should be classified as moderate. The estimated Caco-2 values, which serve as a measurable indicator of oral drug permeability, suggest that most of the considered derivatives exhibit good permeability. The only exceptions to this trend are derivatives **2f** and **3f**, for which the recorded values are slightly below the threshold levels (−5.15 log cm/s) [46]. The Human Intestinal Absorption values for the studied molecules are nearly identical and confirm a very high absorption capacity for all analysed molecules. The hERG value is a crucial test that determines a drug’s ability to inhibit hERG K+ channels [43], which can be a contributing factor in the development of long QT syndrome (LQTS), arrhythmia, and Torsade de Pointes. The presented values indicate that all the studied derivatives, as well as the reference molecule, show a negligible likelihood of such activity. The presented results clearly indicate that within the proposed group of 5-methylisatin derivatives, there are compounds with ADMET characteristics that, in many aspects, surpass those of the reference molecule, particularly in terms of bioavailability (Log*P*, LogS, LogD) and permeability (MDCK). Only in the case of derivatives **2f** and **3f** are slightly lower results observed compared to the reference molecule, particularly regarding passive permeability (Caco-2, MDCK).

### 2.3. Synthesis and NMR Data

**Scheme 1:**
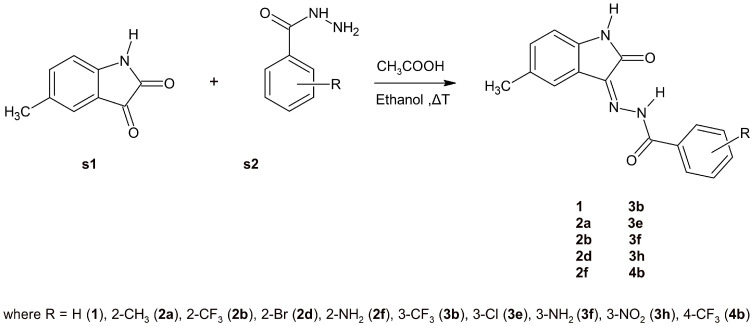
A schematic depiction of the synthesis of the chosen 5-methylisatin-based benzoylhydrazines.

Various methods for the synthesis of 5-methylisatin-based benzoylhydrazines are documented in the literature [32,35,47,48,49]. The simplest approach involves heating 5-methylisatin (**s1**) with substituted benzoylhydrazine (**s2**) in ethanol, using acetic acid as a catalyst (Scheme 1). The structures of the newly synthesised compounds were confirmed by NMR, IR, and elemental analysis.

**Figure 7:**
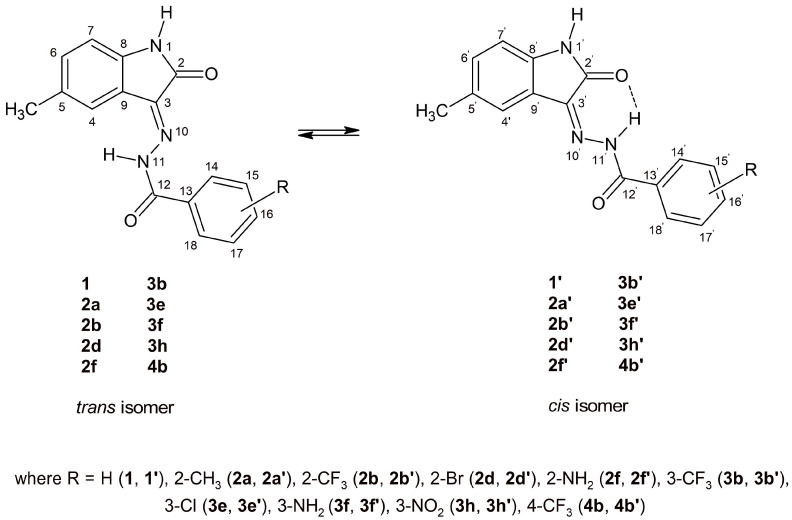
Possible geometry of the synthesised 5-methylisatin-based benzoylhydrazines.

The presence of a double bond between the carbon (C-3) and nitrogen (N-10) atoms in the compounds under study allows for a different spatial arrangement of atoms around the double bond. In addition, the hydrogen atom on the nitrogen N-11 will play a key role in the formation of the hydrogen bond with the oxygen atom of 5-methylisatine [35,50,51]. The aforementioned factors result in the observation of *cis*-*trans* isomerism in the structures of 5-methylisatin-based benzoylhydrazines (Figure 7, Table 6). The investigated structures in the *cis* configuration exhibit greater stability than their *trans* counterparts, which can be attributed to the possibility of forming intramolecular hydrogen bonds. Additionally, in derivatives substituted at position 2 (**2a**, **2b**, **2d**, and **2f**), an intramolecular hydrogen bond was observed between the substituent and the carbonyl oxygen of benzoylhydrazine. According to R.S. Hunoor et al. [51] the N–H group of the hydrazine part is sandwiched between the carbonyl (C=O) and the N=C azomethine nitrogen, forming an intramolecular hydrogen bond with the carbonyl oxygen of 5-methylisatin. These interactions, in accordance with our previous studies [35], present difficulties in the unambiguous assignment of aromatic hydrogens and carbons to individual isomers of 5-methylisatin-based 2-substituted benzoylhydrazines.

Identifying a compound’s structure using nuclear magnetic resonance (NMR) spectroscopy involves interpreting various NMR spectra, primarily ^1^H NMR and ^13^C (including 2D) NMR. NMR spectra clearly indicate the presence of both *cis* and *trans* forms in solution. The most characteristic N–H signals of benzoylhydrazine involved in the relevant intramolecular hydrogen bond for *cis* isomers appear in the range of 13.33–14.06 ppm and 11.46–11.95 ppm for the *trans* form. In accordance with results published earlier for the same type of compounds [35], the ^1^H-NMR signal of N–H of isatin can be seen at δ = 10.71–11.29 ppm and δ = 11.25–11.81 ppm in DMSO, which is a singlet, for *cis* and *trans* isomers, respectively. The singlet at ca. 2.32 ppm indicates a CH_3_ group of 5-methylisatin. Hydrogen atoms bonded to the aromatic ring were observed in the characteristic range for aromatic protons from 6.62 to 8.76 ppm. The chemical shifts in C-12 (ca. 165 ppm) and C-12′ (ca. 163 ppm) for analysed compounds are also comparable to those obtained earlier [35]. The carbon signals of the lactonyl carbon C-2/C-2′ were observed at ca. 142 and 140 ppm, respectively. The chemical shift at approximately 21 ppm in ^13^C NMR spectra, which is typical of carbon atoms in a simple aliphatic (non-aromatic) group, corresponds to the CH_3_ group of 5-methylisatin. This upfield resonance occurs because the methyl carbon is less deshielded compared to carbons that are bonded to more electronegative atoms or are part of more complex structures (e.g., aromatic rings or carbonyl groups).

### 2.4. Infrared Spectral Studies

By analysing the positions and intensities of absorption bands, one can determine the presence of specific functional groups within a molecule. In the IR spectra of 5-methylisatin-based benzoylhydrazine, absorptions related to carbonyl groups of isatin and hydrazide moieties were the most intense ones appearing at 1680–1740 cm^−1^ and 1619–1684 cm^−1^, respectively. Moreover, sharp bands of medium intensity at 3177–3298 cm^−1^ in the spectra of the analysed compounds resulted from stretching vibrations of the –NH group. In accordance with our previous studies [35], the absorption band at 1484–1543 cm^−1^ was also assigned to azomethine ν(C=N) stretching. The characteristic band strength values of the compounds studied are presented in Table 7.

### 2.5. Spectroscopic Properties

**Figure 8:**
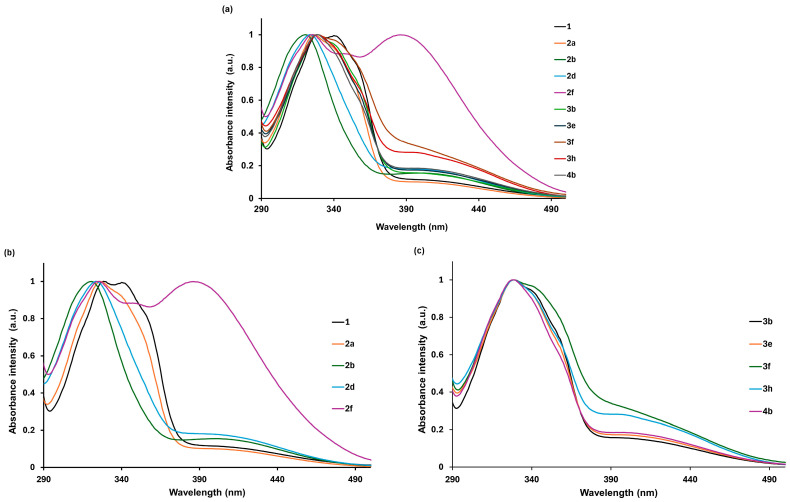
The normalised UV–Vis absorption of the synthesised 5-methylisatin-based benzoylhydrazines in methanol (**a**), the normalised UV–Vis absorption of 2-substituted 5-methylisatin-based benzoylhydrazines in methanol (**b**), and the normalised UV–Vis absorption of 3- or 4-substituted 5-methylisatin-based benzoylhydrazines in methanol (**c**).

The absorption spectra indicate that the compounds under investigation absorb light in the 290–490 nm range (Table 8). The compounds exhibit a single absorption maximum in the 316–342 nm region (Figure 8a). The **2f** derivative (2-NH_2_) is an exception, exhibiting two absorption maxima (the second at approximately 383–386 nm). The shape and position of the absorption band are only slightly dependent on the polarity of the solvent, while the type and position of the substituent have a greater influence. The greatest effect was observed for substituents located in the *ortho* position of hydrazine (Figure 8b,c). The synthesised 5-methylisatin-based benzoylhydrazines are characterised by low molar absorption coefficients (Table 8).

## 3. Materials and Methods

### 3.1. Computational Methods

#### 3.1.1. The Docking Procedure and In Silico ADMET Analysis

The structure of cyclin-dependent kinase 2 (CDK2), identified by PDB ID 1E9H, was sourced from the Brookhaven Protein Database [52]. Initial preparations, including identifying the active site and preparing the structures of the ligands and protein, such as removing all non-polar hydrogen atoms and determining the number of rotatable bonds for the ligands, were carried out using the AutodockTools 1.5.6 package [53]. The grid box was resized to match the active site’s dimensions, which were 16 × 14 × 22 Å. Docking simulations for all ligands were conducted using the Autodock Vina software v1.2.x [54], with the exhaustiveness parameter set to 20 for all simulations. Each ligand underwent the docking procedure five times, each with a different random seed value. The quantitative structure–activity relationship (QSAR) analysis was conducted using ADMETlab 2.0 [40] based on 2D structures described using SMILES. The selected descriptors for molecule evaluation primarily define molecular properties (molecular weight, number of hydrogen bond donors—nHD, number of hydrogen bond acceptors—nHA, Topological Polar Surface Area—TPSA, log*P*, LogD, LogS), absorption (Caco-2 permeability, MDCK permeability, Human Intestinal Absorption—HIA), distribution (blood–brain barrier penetration), and toxicity (hERG Blockers) of the candidates exhibiting the highest binding affinity to the CDK2 active site.

#### 3.1.2. The Molecular Dynamics Simulations

The CDK2 protein structure was characterised using the ff14SB force field parameters [55], while the inhibitor molecules were described using GAFF force field parameters [56]. For the isatin derivatives, charge values were determined using the Merz–Kollmann scheme and the RESP procedure at the HF/6-31G* level [57]. The CDK2–inhibitor complexes were neutralised and immersed in a periodic cubic box containing TIP3P water molecules. During the initial phase, each system was heated to 300 K, with temperature regulation carried out by a Langevin thermostat [58]. Molecular dynamics simulations were run for 80 nanoseconds using the SHAKE algorithm and periodic boundary conditions. Structural analysis, focusing on the stability of interactions sustaining the complexes, was conducted using the VMD 1.9 package [59]. Hydrogen bonds were identified based on the following criteria: a donor–acceptor distance of less than 3.5 Å, a hydrogen–acceptor distance of less than 3 Å, and a donor–hydrogen–acceptor angle greater than 90°. The enthalpic contributions to binding affinity of the isatin derivatives to the CDK2 active site was evaluated using the molecular mechanics Poisson–Boltzmann surface area (MMPBSA) method [60]. The calculations were performed based on 100 randomly selected conformers of the analysed systems from the last 60 ns of the molecular dynamics simulations. The molecular dynamics simulations were performed using the AMBER 14 package [61]. In the data analysis based on mean values and standard deviations for enthalpic contributions to binding affinity and RMSD, statistical analysis was performed using one-way analysis of variance (ANOVA) with the pairwise multiple comparison Holm–Sidak method [62,63], with an overall significance level of α = 0.05.

### 3.2. Materials

All reagents and solvents were purchased from Sigma-Aldrich (Poznań, Poland) and used without further purification. The highest (≥99%) purity of all used chemicals was required for spectroscopic studies.

### 3.3. Synthesis

The general procedure for the synthesis of the *N*′-[5-methyl-2-oxo-1,2-dihydro-3*H*-indol-3-ylidene]benzohydrazide.

Equimolar amounts of 5-methylisatin (**s1**) (0.002 mol) and substituted benzoylhydrazine (**s2**) (0.002 mol) were added to 96% ethanol (50 mL) containing 3 drops of glacial acetic acid. The mixture was heated under reflux for 5 h and then cooled to room temperature. The resulting solid was collected by filtration, washed with cold ethanol and recrystallized from ethanol to give the following compounds (**1**, **2a**, **2b**, **2d**, **2f**, **3b**, **3e**, **3f**, **3h**, and **4h**).

### 3.4. Experimental Measurements

#### 3.4.1. NMR Measurements

The ^1^H NMR spectra were obtained using a Bruker Ascend III spectrometer operating at 400 MHz. Dimethyl sulphoxide served as the solvent, and tetramethylsilane (TMS) was used as the internal standard. Chemical shifts (*δ*) are reported in ppm relative to TMS, with coupling constants (*J*) given in Hz (Figures S3–S22).

#### 3.4.2. Elemental Analysis Measurements

Elemental analysis was performed using a Vario MACRO elemental analyser from Analysensysteme GmbH (Langenselbold, Germany), controlled by VARIOEL software (version 5.14.4.22).

#### 3.4.3. UV-VIS Measurements

The absorption and emission spectra were obtained at room temperature using a 1 cm quartz cuvette. An Agilent Technology UV-Vis Cary 60 Spectrophotometer was used for absorption measurements, while a Hitachi F-7000 Spectrofluorometer was employed for emission spectra.

#### 3.4.4. FTIR Measurements

Infrared spectra were measured using a PerkinElmer FTIR Spectrum Two spectrophotometer with a diamond ATR, spanning the range from 4000 to 400 cm^−1^. Reflectance spectroscopy was utilised for these measurements.

#### 3.4.5. Calorimetric Measurements

The thermal stability of 5-methylisatin derivatives, including the melting point (m.p.) and thermal decomposition temperature (d.t.), was evaluated using a PerkinElmer DSC 6000. Measurements were performed with a heating rate of 10 K/min and a nitrogen flow rate of 20 mL/min to ensure an inert atmosphere. Calibration of the calorimeter was performed using indium and zinc standards, and standard aluminium pans were employed during the measurements.

## 4. Conclusions

Conducted studies and analyses have led to the development of a new group of isatin derivatives exhibiting significant binding capabilities towards the CDK2 enzyme. Utilised molecular modelling methods allowed for the identification of structural modifications in the benzoylhydrazine part that enhance the affinity of the derivatives towards the active site, considering both the type of substituent and its location. It is evident that all compounds selected for synthesis show increased binding capabilities compared to the native molecule, although the scale of observed differences is quite broad. Comparing the affinities of 5-methylisatin derivatives to CDK2 with the results obtained for compounds studied in previous works dedicated to 5-nitroisatin derivatives [35,38], we observe slightly weaker binding capabilities compared to the best representatives of that group. A distinguishing factor of the new group of compounds is that the methyl substituent in the fifth position of the isatin core, unlike the nitro group, does not engage the isatin core in hydrogen bonds with amino acids such as ASP145 or LYS33, which results in deeper embedding of this fragment in the hydrophobic pocket. For almost all analysed derivatives, there is a significant pattern in the fact that the presence of functional groups on the phenyl ring, regardless of identifying potential new interactions, contributes to deeper and tighter embedding of the molecular core in the hydrophobic pocket of the active site. A multidimensional comparative analysis of the proposed 5-methylisatin derivatives against the reference molecule reveals several areas where the newly developed compounds demonstrate significant advantages, particularly in terms of affinity for the active site, as estimated through both docking and molecular dynamics, conformational stability of the complexes, and fit to the active site. The ADMET analysis shows that the proposed compounds perform comparably in most evaluated areas, while in terms of bioavailability molecules **1**, **2a**, **2b**, **3h**, **3b** and **3e** exhibit notable advantages over the reference molecule.

The selected 5-methylisatin-based benzoylhydrazines compounds (**1**, **2a**, **2b**, **2d**, **2f**, **3b**, **3e**, **3f**, **3h** and **4b**) were synthesised with a good yield and their spectroscopic properties were characterised in three solvents of different polarities. The compounds under investigation, similarly to those previously tested [35], exhibited a pronounced absorption in the wavelength range of 295 nm and 490 nm. Nuclear magnetic resonance spectra demonstrated that 5-methylisatin-based benzoylhydrazines in solution exist in both the *cis* and *trans* forms.

After considering all aspects of the studies conducted, including dynamic conformational analysis and energetic characterisation of the complexes through computational chemistry methods, alongside the capability to synthesise and isolate the examined derivatives, it can be concluded that molecules such as **3f**, **3b**, **3h**, **2f**, **2b** and **2a** could potentially serve as candidates for anticancer drugs. Therefore, further research into their biological activity is particularly justified.

## Figures and Tables

**Table 1:** The averaged values of biding affinity and inhibition constants (IC) of 5-methylisatin-based benzoylhydrazides towards the CDK2 active site. The value for native molecule (1) is 9.18 ± 0.04 [kcal/mol]. Values in parentheses represent standard deviation values.

Chemical Group	Name	Binding Affinity [kcal/mol]	IC [nM]	Name	Binding Affinity [kcal/mol]	IC [nM]	Name	Binding Affinity [kcal/mol]	IC [nM]
**-H**	**1**	**−9.18 (0.04)**	**186.63**	---	---	---	---	---	---
**-CH_3_**	**2a**	**−9.60 (*0.00*)**	**91.86**	**3a**	−9.50 (*0.00*)	108.74	**4a**	−9.44 (*0.05*)	120.33
**-CF_3_**	**2b**	**−9.80 (*0.00*)**	**65.54**	**3b**	**−9.84 (*0.05*)**	**61.26**	**4b**	**−9.70 (*0.00*)**	**77.59**
**-F**	**2c**	−9.40 (*0.00*)	128.74	**3c**	−9.50 (*0.00*)	108.74	**4c**	−9.30 (*0.00*)	152.41
**-Br**	**2d**	**−9.58 (*0.04*)**	**95.01**	**3d**	−9.50 (*0.00*)	108.74	**4d**	−9.40 (*0.00*)	128.74
**-Cl**	**2e**	−9.50 **(*0.00*)**	108.74	**3e**	**−9.56 (*0.05*)**	**98.27**	**4e**	−9.32 (*0.04*)	147.35
**-NH_2_**	**2f**	**−9.60 (*0.00*)**	**91.86**	**3f**	**−9.64 (*0.05*)**	**85.86**	**4f**	−9.10 (*0.00*)	213.61
**-N(CH_3_)_2_**	**2g**	−8.30 (*0.00*)	824.17	**3g**	−9.10 (*0.00*)	213.61	**4g**	−9.20 (*0.00*)	180.43
**-NO_2_**	**2h**	−9.00 (*0.00*)	252.88	**3h**	**−9.50 (*0.00*)**	**108.74**	**4h**	−9.24 (*0.05*)	168.65
**-OH**	**2i**	−9.20 (*0.00*)	180.43	**3i**	−9.20 (*0.00*)	180.43	**4i**	−9.10 (*0.00*)	213.61
**-OCH_3_**	**2j**	−8.98 (*0.00*)	261.56	**3j**	−9.20 (*0.00*)	180.43	**4j**	−9.20 (*0.00*)	180.43

**Table 2:** Values of hydrogen bond lengths and distances between aromatic systems involved in the stabilisation of complexes obtained at the docking stage. The designation **RM** refers to the reference molecule 3-((2,6-Dichlorobenzylidene)hydrazono)indolin-2-one.

Name	Distances [Å]				
	GLU 81	LEU 83 H	LEU 83 O	Phe80	Phe82
**1**	2.32	1.83	2.67	4.08	4.06
**2a**	2.15	1.88	3.23	3.57	4.07
**2b**	2.15	1.90	3.42	3.82	4.01
**2d**	2.13	1.85	3.32	3.87	4.06
**2f**	2.21	1.88	2.96	3.58	4.11
**3b**	2.14	1.87	3.28	3.57	4.36
**3e**	2.19	1.86	3.05	3.90	4.08
**3f**	2.24	1.82	2.80	4.04	4.03
**3h**	2.43	1.84	2.53	4.23	3.69
**4b**	2.21	1.84	2.81	3.95	3.88
**RM**	2.41	2.04	----	3.70	5.17

**Table 3:** Averaged RMSD values for ligands and the CDK2 protein for conformers from the 80 ns and the final 60 ns of the molecular dynamics simulation. For values in bold, statistical analysis based on one-way analysis of variance with the pairwise multiple comparison Holm–Sidak method showed no significant statistical differences between the data populations collected during the first 80 ns and the final 60 ns of the MD simulation.

Name	Ligand				CDK2			
	80 ns		Last 60 ns		80 ns		Last 60 ns	
	RMSD	SD	RMSD	SD	RMSD	SD	RMSD	SD
**1**	**0.33**	**0.10**	**0.33**	**0.10**	2.44	0.28	2.56	0.12
**2a**	1.27	0.41	1.45	0.06	2.57	0.36	2.70	0.26
**2b**	**0.89**	**0.17**	**0.91**	**0.16**	2.49	0.40	2.68	0.19
**2d**	**0.45**	**0.16**	**0.45**	**0.17**	**2.27**	**0.19**	**2.29**	**0.15**
**2f**	**0.77**	**0.16**	**0.79**	**0.17**	2.67	0.33	2.77	0.28
**3b**	1.53	0.59	1.82	0.35	2.31	0.31	2.43	0.19
**3e**	**0.63**	**0.22**	**0.65**	**0.22**	2.52	0.34	2.63	0.26
**3f**	**0.25**	**0.07**	**0.24**	**0.06**	2.16	0.17	2.19	0.11
**3h**	**0.66**	**0.15**	**0.67**	**0.15**	2.35	0.30	2.47	0.19
**4a**	0.91	0.29	0.96	0.30	2.41	0.37	2.50	0.34
**RM**	1.16	0.25	1.24	0.12	2.49	0.24	2.58	0.15

**Table 4:** The cumulative analysis of the length of the interactions identified in the CDK2 complexes with the selected 5-methylisatin-based benzoylhydrazines. The distances presented in the table represent middle values of intervals with a width of 0.2 Å.

Interactions	Population %								
	Σ	1.6 Å	1.8 Å	2 Å	2.2 Å	2.4 Å	2.6 Å	2.8 Å	3 Å
**1**									
Ligand (**H1**) … (**O**) GLU 81	100	1.94	48.31	41.44	7.56	0.69	0.06	0.00	0.00
Ligand (**O1**) … (**H**N) LEU 83	100	0.88	26.75	46.13	19.50	5.38	1.25	0.13	0.00
Ligand (**H5**) … (**O**) LEU 83	20.6	0.00	0.19	0.31	0.75	1.25	3.44	4.94	9.69
**2a**									
Ligand (**H1**) … (**O**) GLU 81	100	1.94	48.94	40.94	7.63	0.50	0.06	0.00	0.00
Ligand (**O2**) … (**H**N) LEU 83	99.56	0.25	13.69	30.94	28.56	16.31	5.56	3.00	1.25
**2b**									
Ligand (**H1**) … (**O**) GLU 81	100	1.19	41.31	44.00	11.37	1.81	0.31	0.00	0.00
Ligand (**O1**) … (**H**N) LEU 83	100	1	29	46.8125	18.125	4	0.9375	0.0625	0.0625
Ligand (**H5**) … (**O**) LEU 83	55.5	0.44	10.62	18.56	11.00	4.31	2.62	3.75	4.19
**2d**									
Ligand (**H1**) … (**O**) GLU 81	100	2.25	49.44	41.31	6.37	0.62	0.00	0.00	0.00
Ligand (**O1**) … (**H**N) LEU 83	100	0.12	16.19	39.94	27.94	10.81	3.87	1.00	0.12
**2f**									
Ligand (**H1**) … (**O**) GLU 81	100	2.69	48.00	40.19	8.06	0.81	0.25	0.00	0.00
Ligand (**O1**) … (**H**N) LEU 83	99.94	0.75	29.81	45.25	16.75	5.56	1.44	0.19	0.19
Ligand (**H**) … (**O**) ILE10	62.44	0.13	7.63	15.25	12.38	10.19	6.38	5.00	5.50
Ligand (N**H**) … (**O2**) Ligand	98.13	0.00	7.44	35.81	30.94	14.63	5.69	2.31	1.31
**3b**									
Ligand (**H1**) … (**O**) GLU 81	100.00	1.25	41.31	44.75	10.88	1.63	0.13	0.06	0.00
Ligand (**O1**) … (**H**N) LEU 83	100.00	1.06	30.69	42.13	18.69	5.44	1.56	0.44	0.00
Ligand (**H5**) … (**O**) LEU 83	46.56	0.06	4.44	10.69	7.38	6.19	4.75	5.63	7.44
**3e**									
Ligand (**H3**) … (**O**) GLU 81	100.00	2.00	49.00	41.63	6.50	0.88	0.00	0.00	0.00
Ligand (**O2**) … (**H**N) LEU 83	100.00	0.88	34.19	46.06	15.19	3.06	0.63	0.00	0.00
Ligand (**H5**) … (**O**) LEU 83	39.44	0.00	0.69	2.06	2.88	3.50	6.44	9.56	14.31
**3f**									
Ligand (**H1**) … (**O**) GLU 81	100.00	1.63	43.88	44.19	9.56	0.63	0.13	0.00	0.00
Ligand (**O1**) … (**H**N) LEU 83	100.00	0.25	14.69	44.00	27.69	10.25	2.56	0.44	0.13
Ligand (N**H**) … (**O**) HIE 84	97.06	0.44	14.88	33.94	22.13	11.81	7.19	4.31	2.38
Ligand (N**H**) … (**O**) LEU 83	99.56	0.50	18.13	41.50	25.56	8.94	3.56	1.19	0.19
Ligand (**H5**) … (**O1**) Ligand	100.00	0.00	2.25	23.13	41.38	23.75	7.88	1.38	0.00
**3h**									
Ligand (**H1**) … (**O**) GLU 81	100.00	1.00	42.81	43.88	10.31	1.56	0.44	0.00	0.00
Ligand (**O1**) … (**H**N) LEU 83	100.00	1.00	32.25	45.94	15.75	3.81	0.94	0.13	0.19
Ligand (**H5**) … (**O**) LEU 83	44.13	0.00	0.81	4.50	4.31	7.00	7.56	9.38	10.56
**4b**									
Ligand (**H1**) … (**O**) GLU 81	100.00	2.06	45.63	42.69	8.31	1.13	0.19	0.00	0.00
Ligand (**O1**) … (**H**N) LEU 83	100.00	1.38	32.69	42.38	18.06	4.00	1.25	0.19	0.06
Ligand (**H5**) … (**O**) LEU 83	30.38	0.00	0.81	2.44	3.63	4.31	4.31	5.69	9.19
**RM**									
Ligand (**H1**) … (**O**) GLU 81	99.94	1.31	39.44	44.25	12.94	1.75	0.25	0.00	0.00
Ligand (**O1**) … (**H**N) LEU 83	98.94	0.13	5.69	23.81	27.25	19.75	12.06	7.13	3.13

**Table 5:** ADMET parameters evaluated for synthesised molecules and reference system using ADMETlab 2.0.

Name	MW ^a^	nHD ^b^	nHA ^c^	TPSA ^d^	Log*P* ^e^	LogD ^f^	LogS ^g^	BBB ^h^	Caco-2 ^i^	MDCK ^j^	HIA ^k^	hERG ^l^
**1**	279.10	2	5	70.56	3.088	2.737	−4.669	0.337	−4.893	1.25 × 10^−5^	0.011	0.051
**2a**	293.12	2	5	70.56	3.450	2.840	−5.142	0.270	−4.855	1.11 × 10^−5^	0.005	0.038
**2b**	347.09	2	5	70.56	3.594	2.991	−5.05	0.183	−4.880	1.84 × 10^−5^	0.004	0.026
**2d**	357.01	2	5	70.56	3.613	2.871	−5.339	0.452	−4.804	1.51 × 10^−5^	0.006	0.064
**2f**	294.11	4	6	96.58	3.118	2.591	−5.167	0.867	−5.287	8.09 × 10^−6^	0.008	0.056
**3b**	347.09	2	5	70.56	3.848	3.105	−5.402	0.192	−4.919	1.65 × 10^−5^	0.005	0.058
**3e**	313.06	2	5	70.56	3.777	2.839	−5.205	0.244	−4.841	1.49 × 10^−5^	0.008	0.089
**3f**	294.11	4	6	96.58	2.711	2.153	−4.584	0.761	−5.486	6.13 × 10^−6^	0.009	0.060
**3h**	324.09	2	8	113.7	3.111	2.409	−4.900	0.063	−4.991	2.57 × 10^−5^	0.012	0.300
**4a**	347.09	2	5	70.56	3.881	3.111	−5.512	0.140	−4.880	1.51 × 10^−5^	0.004	0.062
**RM**	317.01	1	4	53.82	4.462	2.795	−6.693	0.793	−4.793	2.09 × 10^−5^	0.003	0.001

^a^ Molecular weight (<500 u). ^b^ Number of hydrogen bond donors (nHD ≤ 5). ^c^ Number of hydrogen bond acceptors (nHA ≤ 10). ^d^ Topological Polar Surface Area (TPSA ≤ 140). ^e^ Log of the octanol/water partition coefficient (optimal 0~3; <5); ^f^ log*P* at physiological pH 7.4 (optimal: 1~3). ^g^ Log of the aqueous solubility (optimal: −4~0.5). ^h^ Blood–brain barrier penetration; the value is the probability of being BBB+. ^i^ Caco-2 permeability (optimal: higher than −5.15 log cm/s). ^j^ MDCK permeability (low permeability: <2 × 10^−6^ cm/s, medium permeability: 2–20 × 10^−6^ cm/s, high passive permeability: >20 × 10^−6^ cm/s). ^k^ Human Intestinal Absorption, Category 1: HIA+(HIA < 30%); Category 0: HIA-(HIA < 30%). The output value is the probability of being HIA+. ^l^ The output value is the probability of being an active blocker of hERG K+ channels.

**Table 6:** Content of *cis* isomer (%) and the diastereomeric ratio [K] in DMSO-*d_6_* at 296.9 K ^a^.

No.	Substituent	*cis* Isomer (%)	$K=\frac{\mathrm{cis}\mathrm{isomer}}{\mathrm{trans}\mathrm{isomer}}$
**1**	H	100	-
**2a**	2-CH_3_	100	-
**2b**	2-CF_3_	22	0.28
**2d**	2-Br	28	0.39
**2f**	2-NH_2_	49	0.96
**3b**	3-CF_3_	61	1.56
**3e**	3-Cl	49	0.96
**3f**	3-NH_2_	69	2.23
**3h**	3-NO_2_	43	0.75
**4b**	4-CF_3_	44	0.78

^a^ Based on the integral of the H1 signals.

**Table 7:** Characteristic IR bands (cm^−1^) of the synthesised 5-methylisatin-based benzoylhydrazines.

Compound	ν(–NH_2_)		ν(N–H)	ν(C=O)		ν(C=N)	ν(N=O)
	ν_asym_	ν_sym_		Lactam	Hydrazide		
**1**	-	-	3247	1697	1674	1525	1480
**2a**	-	-	3254	1683	1628	1524	1482
**2b**	-	-	3265	1727	1684	1512	1314
**2d**	-	-	3200	1740	1619	1500	1291
**2f**	3483	3347	3177	1716	1627	1487	1318
**3b**	-	-	3282	1714	1674	1498	1330
**3e**	-	-	3278	1731	1677	1484	1321
**3f**	3449	3367	3298	1680	1622	1543	1314
**3h**	-	-	3285	1731	1678	1532	1347
**4b**	-	-	3247	1728	1662	1483	1315

**Table 8:** Spectroscopic properties of the synthesised 5-methylisatin-based benzoylhydrazines in solvents of different polarity.

No.	Substituent	Solvent	*λ_ab_* (nm)	*ε* (×10^4^, M^−1^·cm^−1^)
**1**	H	DCM	*345*	*1.51*
		MeOH	328	2.16
		MeCN	327	2.02
**2a**	2-CH_3_	DCM	340	1.38
		MeOH	326	1.84
		MeCN	324	1.94
**2b**	2-CF_3_	DCM	316	1.76
		MeOH	320	1.63
		MeCN	316	1.65
**2d**	2-Br	DCM	320	1.11
		MeOH	324	1.36
		MeCN	319	1.73
**2f**	2-NH_2_	DCM	324, 383	1.10, 1.10
		MeOH	325, 386	1.67, 1.66
		MeCN	322, 387	1.95, 1.92
**3b**	3-CF_3_	DCM	327	1.86
		MeOH	326	1.42
		MeCN	328	1.83
**3e**	3-Cl	DCM	327	1.70
		MeOH	329	1.63
		MeCN	325	1.70
**3f**	3-NH_2_	DCM	346	1.32
		MeOH	328	2.09
		MeCN	325	1.08
**3h**	3-NO_2_	DCM	325	1.53
		MeOH	329	1.46
		MeCN	324	1.18
**4b**	4-CF_3_	DCM	325	1.64
		MeOH	328	1.32
		MeCN	325	1.57

## Data Availability

All data are placed in the article and supplementary materials.

## Supplementary Materials

The supporting information can be downloaded at https://www.mdpi.com/article/10.3390/ijms26052144/s1.

## References

[1] Boire A., Burke K., Cox T.R., Guise T., Jamal-Hanjani M., Janowitz T., Kaplan R., Lee R., Swanton C., Vander Heiden M.G. (2024). Why do patients with cancer die?. Nat. Rev. Cancer.

[2] Hjartåker A., Weiderpass E., Bray F. (2017). Cancer Mortality. International Encyclopedia of Public Health.

[3] Morgan D.O. (1997). CYCLIN-DEPENDENT KINASES: Engines, Clocks, and Microprocessors. Annu. Rev. Cell Dev. Biol..

[4] Malumbres M., Barbacid M. (2009). Cell cycle, CDKs and cancer: A changing paradigm. Nat. Rev. Cancer.

[5] Besson A., Dowdy S.F., Roberts J.M. (2008). CDK Inhibitors: Cell Cycle Regulators and Beyond. Dev. Cell.

[6] Ghafouri-Fard S., Khoshbakht T., Hussen B.M., Dong P., Gassler N., Taheri M., Baniahmad A., Dilmaghani N.A. (2022). A review on the role of cyclin dependent kinases in cancers. Cancer Cell Int..

[7] Ferraz de Paiva R.E., Vieira E.G., Rodrigues da Silva D., Wegermann C.A., Costa Ferreira A.M. (2021). Anticancer Compounds Based on Isatin-Derivatives: Strategies to Ameliorate Selectivity and Efficiency. Front. Mol. Biosci..

[8] Lashen A., Alqahtani S., Shoqafi A., Algethami M., Jeyapalan J.N., Mongan N.P., Rakha E.A., Madhusudan S. (2024). Clinicopathological Significance of Cyclin-Dependent Kinase 2 (CDK2) in Ductal Carcinoma In Situ and Early-Stage Invasive Breast Cancers. Int. J. Mol. Sci..

[9] Sabnis R.W. (2020). Novel CDK2 Inhibitors for Treating Cancer. ACS Med. Chem. Lett..

[10] Chenette E.J. (2010). A key role for CDK2. Nat. Rev. Cancer.

[11] Sabt A., Eldehna W.M., Al-Warhi T., Alotaibi O.J., Elaasser M.M., Suliman H., Abdel-Aziz H.A. (2020). Discovery of 3,6-disubstituted pyridazines as a novel class of anticancer agents targeting cyclin-dependent kinase 2: Synthesis, biological evaluation and in silico insights. J. Enzym. Inhib. Med. Chem..

[12] Davies T.G., Bentley J., Arris C.E., Boyle F.T., Curtin N.J., Endicott J.A., Gibson A.E., Golding B.T., Griffin R.J., Hardcastle I.R. (2002). Structure-based design of a potent purine-based cyclin-dependent kinase inhibitor. Nat. Struct. Biol..

[13] Coxon C.R., Anscombe E., Harnor S.J., Martin M.P., Carbain B., Golding B.T., Hardcastle I.R., Harlow L.K., Korolchuk S., Matheson C.J. (2017). Cyclin-Dependent Kinase (CDK) Inhibitors: Structure–Activity Relationships and Insights into the CDK-2 Selectivity of 6-Substituted 2-Arylaminopurines. J. Med. Chem..

[14] De Azevedo W.F., Leclerc S., Meijer L., Havlicek L., Strnad M., Kim S. (1997). Inhibition of Cyclin-Dependent Kinases by Purine Analogues. Eur. J. Biochem..

[15] Jorda R., Havlíček L., McNae I.W., Walkinshaw M.D., Voller J., Šturc A., Navrátilová J., Kuzma M., Mistrík M., Bártek J. (2011). Pyrazolo[4,3-*d*]pyrimidine Bioisostere of Roscovitine: Evaluation of a Novel Selective Inhibitor of Cyclin-Dependent Kinases with Antiproliferative Activity. J. Med. Chem..

[16] Liu J.-J., Daniewski I., Ding Q., Higgins B., Ju G., Kolinsky K., Konzelmann F., Lukacs C., Pizzolato G., Rossman P. (2010). Pyrazolobenzodiazepines: Part I. Synthesis and SAR of a potent class of kinase inhibitors. Bioorg. Med. Chem. Lett..

[17] Brasca M.G., Amboldi N., Ballinari D., Cameron A., Casale E., Cervi G., Colombo M., Colotta F., Croci V., D’Alessio R. (2009). Identification of *N*,1,4,4-Tetramethyl-8-{[4-(4-methylpiperazin-1-yl)phenyl]amino}-4,5-dihydro-1 *H*-pyrazolo[4,3-*h*]quinazoline-3-carboxamide (PHA-848125), a Potent, Orally Available Cyclin Dependent Kinase Inhibitor. J. Med. Chem..

[18] Finlay M.R.V., Acton D.G., Andrews D.M., Barker A.J., Dennis M., Fisher E., Graham M.A., Green C.P., Heaton D.W., Karoutchi G. (2008). Imidazole piperazines: SAR and development of a potent class of cyclin-dependent kinase inhibitors with a novel binding mode. Bioorg. Med. Chem. Lett..

[19] Lin S.-F., Lin J.-D., Hsueh C., Chou T.-C., Wong R.J. (2018). Activity of roniciclib in medullary thyroid cancer. Oncotarget.

[20] Ghosh S., Ramarao T.A., Samanta P.K., Jha A., Satpati P., Sen A. (2021). Triazole based isatin derivatives as potential inhibitor of key cancer promoting kinases- insight from electronic structure, docking and molecular dynamics simulations. J. Mol. Graph. Model..

[21] Shin E.-K., Kim J.-K. (2012). Indirubin derivative E804 inhibits angiogenesis. BMC Cancer.

[22] Bramson H.N., Holmes W.D., Hunter R.N., Lackey K.E., Lovejoy B., Luzzio M.J., Montana V., Rocque W.J., Rusnak D., Shewchuk L. (2001). Oxindole-based inhibitors of cyclin-dependent kinase 2 (CDK2): Design, synthesis, enzymatic activities, and X-ray crystallographic analysis. J. Med. Chem..

[23] Czeleń P. (2017). Inhibition mechanism of CDK-2 and GSK-3β by a sulfamoylphenyl derivative of indoline—A molecular dynamics study. J. Mol. Model..

[24] Czeleń P., Szefler B. (2015). Molecular dynamics study of the inhibitory effects of ChEMBL474807 on the enzymes GSK-3β and CDK-2. J. Mol. Model..

[25] Czeleń P. (2016). Molecular dynamics study on inhibition mechanism of CDK-2 and GSK-3β by CHEMBL272026 molecule. Struct. Chem..

[26] Bharathi Dileepan A.G., Daniel Prakash T., Ganesh Kumar A., Shameela Rajam P., Violet Dhayabaran V., Rajaram R. (2018). Isatin based macrocyclic Schiff base ligands as novel candidates for antimicrobial and antioxidant drug design: In vitro DNA binding and biological studies. J. Photochem. Photobiol. B Biol..

[27] Guo H. (2019). Isatin derivatives and their anti-bacterial activities. Eur. J. Med. Chem..

[28] Zhang M.-Z., Chen Q., Yang G.-F. (2015). A review on recent developments of indole-containing antiviral agents. Eur. J. Med. Chem..

[29] Cheke R.S., Patil V.M., Firke S.D., Ambhore J.P., Ansari I.A., Patel H.M., Shinde S.D., Pasupuleti V.R., Hassan M.I., Adnan M. (2022). Therapeutic Outcomes of Isatin and Its Derivatives against Multiple Diseases: Recent Developments in Drug Discovery. Pharmaceuticals.

[30] Nath R., Pathania S., Grover G., Akhtar M.J. (2020). Isatin containing heterocycles for different biological activities: Analysis of structure activity relationship. J. Mol. Struct..

[31] Elsaman T., Mohamed M.S., Eltayib E.M., Abdel-aziz H.A., Abdalla A.E., Munir M.U., Mohamed M.A. (2022). Isatin derivatives as broad-spectrum antiviral agents: The current landscape. Med. Chem. Res..

[32] Abo-Ashour M.F., Eldehna W.M., Nocentini A., Ibrahim H.S., Bua S., Abou-Seri S.M., Supuran C.T. (2018). Novel hydrazido benzenesulfonamides-isatin conjugates: Synthesis, carbonic anhydrase inhibitory activity and molecular modeling studies. Eur. J. Med. Chem..

[33] Cheke R.S., Firke S.D., Patil R.R., Bari S.B. (2018). ISATIN: New Hope Against Convulsion. Cent. Nerv. Syst. Agents Med. Chem..

[34] Cheng W., Yang Z., Wang S., Li Y., Wei H., Tian X., Kan Q. (2019). Recent development of CDK inhibitors: An overview of CDK/inhibitor co-crystal structures. Eur. J. Med. Chem..

[35] Czeleń P., Skotnicka A., Szefler B. (2022). Designing and Synthesis of New Isatin Derivatives as Potential CDK2 Inhibitors. Int. J. Mol. Sci..

[36] Czeleń P., Szefler B. (2021). The Oxindole Derivatives, New Promising GSK-3β Inhibitors as One of the Potential Treatments for Alzheimer’s Disease—A Molecular Dynamics Approach. Biology.

[37] Czeleń P. (2019). Investigation of the Inhibition Potential of New Oxindole Derivatives and Assessment of Their Usefulness for Targeted Therapy. Symmetry.

[38] Czeleń P., Jeliński T., Skotnicka A., Szefler B., Szupryczyński K. (2023). ADMET and Solubility Analysis of New 5-Nitroisatine-Based Inhibitors of CDK2 Enzymes. Biomedicines.

[39] Al-Salem H.S., Arifuzzaman M., Alkahtani H.M., Abdalla A.N., Issa I.S., Alqathama A., Albalawi F.S., Rahman A.F.M.M. (2020). A Series of Isatin-Hydrazones with Cytotoxic Activity and CDK2 Kinase Inhibitory Activity: A Potential Type II ATP Competitive Inhibitor. Molecules.

[40] Xiong G., Wu Z., Yi J., Fu L., Yang Z., Hsieh C., Yin M., Zeng X., Wu C., Lu A. (2021). ADMETlab 2.0: An integrated online platform for accurate and comprehensive predictions of ADMET properties. Nucleic Acids Res..

[41] Daina A., Michielin O., Zoete V. (2017). SwissADME: A free web tool to evaluate pharmacokinetics, drug-likeness and medicinal chemistry friendliness of small molecules. Sci. Rep..

[42] Lei T., Li Y., Song Y., Li D., Sun H., Hou T. (2016). ADMET evaluation in drug discovery: 15. Accurate prediction of rat oral acute toxicity using relevance vector machine and consensus modeling. J. Cheminform..

[43] Wang S., Sun H., Liu H., Li D., Li Y., Hou T. (2016). ADMET Evaluation in Drug Discovery. 16. Predicting hERG Blockers by Combining Multiple Pharmacophores and Machine Learning Approaches. Mol. Pharm..

[44] Lipinski C.A., Lombardo F., Dominy B.W., Feeney P.J. (2001). Experimental and computational approaches to estimate solubility and permeability in drug discovery and development settings. Adv. Drug Deliv. Rev..

[45] Prasanna S., Doerksen R.J. (2009). Topological Polar Surface Area: A Useful Descriptor in 2D-QSAR. Curr. Med. Chem..

[46] Wang N.-N., Dong J., Deng Y.-H., Zhu M.-F., Wen M., Yao Z.-J., Lu A.-P., Wang J.-B., Cao D.-S. (2016). ADME Properties Evaluation in Drug Discovery: Prediction of Caco-2 Cell Permeability Using a Combination of NSGA-II and Boosting. J. Chem. Inf. Model..

[47] Katiyar A., Hegde M., Kumar S., Gopalakrishnan V., Bhatelia K.D., Ananthaswamy K., Ramareddy S.A., De Clercq E., Choudhary B., Schols D. (2015). Synthesis and evaluation of the biological activity of N′-[2-oxo-1,2 dihydro-3H-indol-3-ylidene] benzohydrazides as potential anticancer agents. RSC Adv..

[48] Debnath K., Pathak S., Pramanik A. (2013). Facile synthesis of ninhydrin and isatin based hydrazones in water using PEG-OSO3H as a highly efficient and homogeneous polymeric acid-surfactant combined catalyst. Tetrahedron Lett..

[49] Haj Mohammad Ebrahim Tehrani K., Hashemi M., Hassan M., Kobarfard F., Mohebbi S. (2016). Synthesis and antibacterial activity of Schiff bases of 5-substituted isatins. Chin. Chem. Lett..

[50] Emami S., Valipour M., Kazemi Komishani F., Sadati-Ashrafi F., Rasoulian M., Ghasemian M., Tajbakhsh M., Honarchian Masihi P., Shakiba A., Irannejad H. (2021). Synthesis, in silico, in vitro and in vivo evaluations of isatin aroylhydrazones as highly potent anticonvulsant agents. Bioorg. Chem..

[51] Hunoor R.S., Patil B.R., Badiger D.S., Chandrashekhar V.M., Muchchandi I.S., Gudasi K.B. (2015). Co(II), Ni(II), Cu(II) and Zn(II) complexes of isatinyl-2-aminobenzoylhydrazone: Synthesis, characterization and anticancer activity. Appl. Organomet. Chem..

[52] Davies T.G., Tunnah P., Meijer L., Marko D., Eisenbrand G., Endicott J.A., Noble M.E. (2001). Inhibitor binding to active and inactive CDK2: The crystal structure of CDK2-cyclin A/indirubin-5-sulphonate. Structure.

[53] Bartashevich E.V., Potemkin V.A., Grishina M.A., Belik A.V. (2002). A Method for Multiconformational Modeling of the Three-Dimensional Shape of a Molecule. J. Struct. Chem..

[54] Trott O., Olson A.J. (2009). AutoDock Vina: Improving the speed and accuracy of docking with a new scoring function, efficient optimization, and multithreading. J. Comput. Chem..

[55] Maier J.A., Martinez C., Kasavajhala K., Wickstrom L., Hauser K.E., Simmerling C. (2015). ff14SB: Improving the Accuracy of Protein Side Chain and Backbone Parameters from ff99SB. J. Chem. Theory Comput..

[56] Wang J., Wolf R.M., Caldwell J.W., Kollman P.A., Case D.A. (2004). Development and testing of a general amber force field. J. Comput. Chem..

[57] Bayly C.I., Cieplak P., Cornell W., Kollman P.A. (1993). A well-behaved electrostatic potential based method using charge restraints for deriving atomic charges: The RESP model. J. Phys. Chem..

[58] Adelman S.A. (1976). Generalized Langevin equation approach for atom/solid-surface scattering: General formulation for classical scattering off harmonic solids. J. Chem. Phys..

[59] Humphrey W., Dalke A., Schulten K. (1996). VMD: Visual molecular dynamics. J. Mol. Graph..

[60] Miller B.R., McGee T.D., Swails J.M., Homeyer N., Gohlke H., Roitberg A.E. (2012). *MMPBSA.py*: An Efficient Program for End-State Free Energy Calculations. J. Chem. Theory Comput..

[61] Case D.A., Babin V., Berryman J.T., Betz R.M., Cai Q., Cerutti D.S., Cheatham T.E., Darden T.A., Duke R.E., Gohlke H. AMBER 14 2014, University of California, San Francisco. https://orbilu.uni.lu/handle/10993/16614.

[62] Kelava A., Moosbrugger H., Dimitruk P., Schermelleh-Engel K. (2008). Methodology: European Journal of Research Methods for the Behavioral and Social Sciences. Methodology.

[63] Stoline M.R. (1981). The Status of Multiple Comparisons: Simultaneous Estimation of All Pairwise Comparisons in One-Way ANOVA Designs. Am. Stat..

