# Supplementary material for: Design and Synthesis of New 5-Methylisatin Derivatives as Potential CDK2 Inhibitors
Source: Int J Mol Sci. 2025 Feb 27;26(5):2144. doi: 10.3390/ijms26052144 (PMC11900410; doi:10.3390/ijms26052144)
Supplement: Supplementary file 1 [file ijms-26-02144-s001.zip › ijms-3488386-supplementary.pdf]

Designing and synthesis of new 5 methyl-Izatin derivatives as potential CDK2 inhibitors

Przemysław Czeleń [1*], Agnieszka Skotnicka [2], Beata Szefler [1], Janina Kabatc[2], Paweł Sutkowy[3]

[1]Department of Physical Chemistry, Faculty of Pharmacy, Collegium Medicum, Nicolaus Copernicus University, Kurpinskiego 5, 85-096 Bydgoszcz, Poland

[2]Faculty of Chemical Technology and Engineering, UTP University of Science and Technology, Seminaryjna 3, 85-326 Bydgoszcz, Poland

[3]Department of Medical Biology and Biochemistry, Faculty of Medicine, Collegium Medicum, Nicolaus Copernicus University, Karłowicza 24, 85-092 Bydgoszcz, Poland

The elemental analysis 2

Figure S1. Distributions of RMSD values. Black distributions refer to ligand molecules while red distributions refer to CDK-2 protein 4

Figure S2. The graphic representation of complexes illustrating conformational changes observed for derivative 2a (a, b) and derivative 3b (c, d). 5

Figure S3. 1H NMR spectrum (400 MHz) of 1 in DMSO-d6. 6

Figure S4. 13C NMR spectrum (100 MHz) of 1 in DMSO-d6. 6

Figure S5. 1H NMR spectrum (400 MHz) of 2a in DMSO-d6. 7

Figure S6. ^{13}C NMR spectrum (100 MHz) of 2a in DMSO-d_6. 7

Figure S7. ^{1}H NMR spectrum (400 MHz) of 2b in DMSO-d_6. 8

Figure S8. ^{13}C NMR spectrum (100 MHz) of 2b in DMSO-d_6. 8

Figure S9. ^{1}H NMR spectrum (400 MHz) of 2d in DMSO-d_6. 9

Figure S10. 13C NMR spectrum (100 MHz) of 2d in DMSO-d6. 9

Figure S11. 1H NMR spectrum (400 MHz) of 2f in DMSO-d6. 10

Figure S12. 13C NMR spectrum (100 MHz) of 2f in DMSO-d6. 10

Figure S13. 1H NMR spectrum (400 MHz) of 3b in DMSO-d6. 11

Figure S14. 13C NMR spectrum (100 MHz) of 3b in DMSO-d6. 11

Figure S15. 1 H NMR spectrum (400 MHz) of 3e in DMSO-d6. 12

Figure S16. 13C NMR spectrum (100 MHz) of 3e in DMSO-d6. 12

Figure S17. 1H NMR spectrum (400 MHz) of 3f in DMSO-d6. 13

Figure S18. 13C NMR spectrum (100 MHz) of 3f in DMSO-d6. 13

Figure S19. 1H NMR spectrum (100 MHz) of 3f in DMSO-d6. 14

Figure S20. 13C NMR spectrum (100 MHz) of 3f in DMSO-d6. 14

Figure S21. 1H NMR spectrum (400 MHz) of 4b in DMSO-d6. 15

Figure S22. 13C NMR spectrum (100 MHz) of 4b in DMSO-d6. 15

The elemental analysis

N'-[5-methyl-2-oxo-1,2-dihydro-3*H*-indol-3-ylidene]benzohydrazide (1) Orange solid, yield 89%, d.t. 284.0 °C, IR (ATR), cm^{-1}: 3247, 1697, 1674, 1525, 1376. ^1H NMR (DMSO-d_6 from TMS) δ (ppm) for *cis*-isomer (1′): 13.96 (s, 1H), 11.29 (s, 1H), 7.90 (m, 2H), 7.69 (m, 1H), 7.62 (t, 1H), 7.44 (s, 1H), 7.21 (d, *J* = 7.92 Hz, 1H), 6.86 (d, *J* = 7.92 Hz, 1H), 2.32 (s, 3H). ^{13}C NMR δ (ppm) for *cis*-isomer (1′): 163.6, 140.7, 138.7, 133.3, 132.7, 132.6, 132.4, 129.6, 127.9, 121.8, 120.3, 111.5, 21.00. $C_{16}H_{13}N_3O_2$, Calcd. C, 68.81, H 4.69, N, 15.05. Found C, 68.50, H 4.80, N, 15.25.

N'-[5-methyl-2-oxo-1,2-dihydro-3*H*-indol-3-ylidene]-2-methylbenzohydrazide (2a) Orange solid, yield 85%, d.t. 218.3 °C, IR (ATR), cm^{-1}: 3254, 1683, 1628, 1524, 1482. ^1H NMR (DMSO-d_6 from TMS) δ (ppm) for *cis*-isomer (2a′): 13.38 (s, 1H), 11.21 (s, 1H), 7.57 (s, 1H), 7.41 (m, 4H), 7.20 (d, *J* = 7.68 Hz, 1H), 6.84 (d, *J* = 7.92 Hz, 1H), 2.34 (s, 3H), 2.30 (s, 3H). ^{13}C NMR δ (ppm) for *cis*-isomer (2a′): 163.3, 140.7, 137.3, 133.8, 132.7, 132.3, 131.5, 127.8, 126.5, 121.6, 120.3, 111.4, 21.00, 20.10. $C_{17}H_{15}N_3O_2$, Calcd. C, 69.61, H, 5.15, N, 14.33. Found C, 69.76, H, 5.10, N, 14.23, O.

N'-[5-methyl-2-oxo-1,2-dihydro-3*H*-indol-3-ylidene]-2-(trifluoromethyl)benzohydrazide (2b) Dark yellow solid, yield 54%, d.t. 295 °C, IR (ATR), cm^{-1}: 3265, 1727 1684, 1512, 1314. ^1H NMR (DMSO-d_6 from TMS) δ (ppm) for *cis*- (2b′) and *trans*-isomer (2b): 13.35 (s, 1H), 13.10 (s, 1H), 11.81 (s, 1H), 11.26 (s, 1H), 10.71 (d, *J* = 56 Hz 1H), 8.10 (s, 1H), 7.82 (m, 7H), 7.58 (s, 1H), 7.46 (s, 1H), 7.21 (d, *J* = 7.92 Hz, 1H), 7.15 (d, *J* = 8.04 Hz, 1H), 6.83 (m, 2H), 2.26 (m, 6H). ^{13}C NMR δ (ppm) for *cis*- (2b) and *trans*-isomer (2b′): 142.3, 134.4, 133.6, 132.8, 131.1, 129.4, 129.2, 128.3, 127.4, 126.9, 125.6, 122.8, 115.7, 111.6, 110.8, 21.00. $C_{17}H_{12}F_3N_3O_2$, Calcd. C, 58.79, H, 3.48, N, 12.10. Found C, 58.63, H, 3.28, N, 12.36.

N'-[5-methyl-2-oxo-1,2-dihydro-3*H*-indol-3-ylidene]-2-bromobenzohydrazide (2d) Orange solid, yield 83%, d.t. 209.7 °C, IR (ATR), cm^{-1}: 3200, 1740 1619, 1500, 1291. ^1H NMR (DMSO-d_6 from TMS) δ (ppm) for *cis*- (2d′) and *trans*-isomer (2d): 13.33 (s, 1H), 13.06 (s, 1H), 11.77 (s, 1H), 11.25 (s, 1H), 10.70 (s, 1H), 8.08 (s, 1H), 7.86 (s, 1H), 7.76 (m, 2H), 7.45 (m, 5H), 7.20 (d, *J* = 7.88 Hz, 2H), 6.91 (s, 1H), 6.83 (m, 1H), 6.78 (d, *J* = 7.88 Hz, 1H), 2.27 (m, 6H). ^{13}C NMR δ (ppm) for *cis*- (2d′) and *trans*-isomer (2d): 165.0, 142.1, 140.8, 137.5, 136.1, 134.1, 133.7, 133.1, 132.1, 131.1, 130.00, 128.8, 128.1, 127.4, 122.00, 120.0, 119.7, 119.7, 115.7, 111.6, 110.8, 21.00. $C_{16}H_{12}BrN_3O_2$, Calcd. C, 53.65, H, 3.38, N, 11.73. Found C, 53.80, H, 3.45, N, 11.52.

N'-[5-methyl-2-oxo-1,2-dihydro-3*H*-indol-3-ylidene]-2-aminobenzohydrazide (2f) Orange solid, yield 96%, d.t. 278.0 °C, IR (ATR), cm^{-1}: 3483, 3347, 3177, 1716, 1627, 1487, 1318. ^1H NMR (DMSO-d_6 from TMS) δ (ppm) for *cis*- (2f′) and *trans*-isomer (2f): 13.85 (s, 1H), 11.25 (s, 1H), 10.71 (s, 1H), 7.74 (s, 1H), 7.68 (m, 1H), 7.44 (m, 1H), 7.29 (m, 2H), 7.20 (m, 2H), 6.84 (m, 5H), 6.76 (bs, 2H), 6.66 (m, 3H), 2.32 (s, 6H). ^{13}C NMR δ (ppm) for *cis*- (2f′) and *trans*-isomer (2f): 167.6, 165.3, 163.6, 151.7, 150.6, 141.9, 141.1, 140.4, 137.6, 134.0, 133.5, 133.3, 132.3, 132.2, 131.0, 130.00, 127.6, 127.1, 121.5, 120.5, 117.7, 117.5, 116.3, 116.0, 115.7, 114.1, 111.7, 111.4, 110.8, 21.2, 21.0. $C_{16}H_{14}N_4O_2$, Calcd. C, 65.30, H, 4.79, N, 19.04. Found C, 65.48, H, 4.69 N, 18.96.

N'-[5-methyl-2-oxo-1,2-dihydro-3*H*-indol-3-ylidene]-3-(trifluoromethyl)benzohydrazide (3b) Light orange, yield 87%, d.t. 308.8 °C, IR (ATR), cm^{-1}: 3282, 1714, 1674, 1498, 1330. ^1H NMR (DMSO-d_6 from TMS) δ (ppm) for *cis*- (3b′) and *trans*-isomer (3b): 14.01 (s, 1H), 11.84 (s, 1H), 11.29 (s, 1H), 10.76 (s, 1H), 8.27 (s, 1H), 8.25 (s, 1H), 8.18 (s, 1H), 8.16 (s, 1H), 8.07 (d, *J* = 7.42 Hz, 1H), 8.03 (d, *J* = 7.84 Hz, 1H), 7.85 (m, 2H), 7.44 (m, 1H), 7.23 (m, 3H), 6.87 (d, *J* = 7.92 Hz, 1H), 6.82 (d, *J* = 7.92 Hz, 1H), 2.31 (s, 6H). ^{13}C NMR δ (ppm) for *cis*- (3b′) and *trans*-isomer (3b): 165.1, 163.5, 142.4, 140.9, 134.4, 133.9, 133.7, 133.12, 133.00, 132.4, 131.1, 131.00, 130.3, 129.8, 129.7, 129.5, 129.1, 128.3, 127.8, 125.7, 125.6, 123.0, 122.7, 121.9, 120.2, 116.0, 111.6, 110.9, 21.00. $C_{17}H_{12}F_3N_3O_2$, Calcd. C, 58.79, H, 3.48, N, 12.10. Found C, 58.55, H, 3.60, N, 12.22.

N'-[5-methyl-2-oxo-1,2-dihydro-3*H*-indol-3-ylidene]-3-chlorobenzohydrazide (3e) Orange solid, yield 95%, d.t. 293.2 °C, IR (ATR), cm^{-1}: 3278, 1731, 1677, 1484, 1321. ^1H NMR (DMSO-d_6 from TMS) δ (ppm) for *cis*- (3e′) and *trans*-isomer (3e): 13.92 (s, 1H), 11.73 (s, 1H), 11.30 (s, 1H), 10.75 (s, 1H), 7.99 (s, 1H), 7.93 (s, 1H), 7.90 (m, 1H), 7.84 (m, 1H), 7.82 (m, 1H), 7.80 (s, 1H), 7.78 (m, 1H), 7.76 (m, 1H), 7.74 (m, 1H), 7.72 (m, 1H), 7.66 (t, 1H), 7.62 (t, 1H), 7.44 (s, 1H), 7.22 (t, 2H), 6.86 (d, *J* = 7.92 Hz, 1H), 6.82 (d, *J* = 7.96 Hz, 1H), 2.31 (s, 6H). ^{13}C NMR δ (ppm) for *cis*- (3e′) and *trans*-isomer (3e): 165.1, 163.5, 142.3, 140.8, 135.5, 134.7, 134.3, 133.8, 133.7, 133.0, 132.9, 132.4, 131.6, 131.1, 131.00, 128.8, 127.8, 127.6, 126.5, 121.9, 120.2, 116.1, 111.6, 110.9, 21.1, 21.0. $C_{16}H_{12}ClN_3O_2$ Calcd. C, 61.25, H, 3.86, N, 13.39. Found C, 61.15, H, 3.78, N, 13.57.

N'-[5-methyl-2-oxo-1,2-dihydro-3*H*-indol-3-ylidene]-3-aminobenzohydrazide (3f) Orange solid, yield 91%, d.t. 270.5 °C, IR (ATR), cm^{-1}: 3449, 3367, 3298, 1680, 1622, 1543, 1314. ^1H NMR (DMSO-d_6 from TMS) δ (ppm) for *cis*- (3f′) and *trans*-isomer (3f): 13.84 (s, 1H), 11.46 (s, 1H), 11.26 (s, 1H), 10.72 (s, 1H), 7.69 (s, 1H), 7.43 (s, 1H), 7.21 (m, 4H), 7.13 (m, 2H), 7.08 (d, *J* = 7.68 Hz, 1H), 6.96 (d, *J* = 7.92 Hz, 1H), 6.87 (s, 1H), 6.83 (m, 3H), 5.52 (s, 4H) 2.32 (s, 6H). ^{13}C NMR δ (ppm) for *cis*- (3f′) and *trans*-isomer (3f): 167.0, 165.3, 164.0, 164.0, 15.00, 149.5, 142.1, 142.0, 140.6, 138.3, 134.1, 133.5, 133.3, 132.6, 132.3, 131.0, 130.0, 129.6, 127.5, 121.7, 120.4, 118.8, 118.0, 116.3, 115.8, 114.3, 113.9, 113, 111.4, 110.9, 21.2, 21.0. $C_{16}H_{14}N_4O_2$, Calcd. C, 65.30, H, 4.79, N, 19.04. Found C, 65.25, H, 4.84 N, 19.04.

N'-[5-methyl-2-oxo-1,2-dihydro-3*H*-indol-3-ylidene]-3-nitrobenzohydrazide (3h) Orange solid, yield 92%, d.t. 313.9 °C, IR (ATR), cm^{-1}: 3285, 1731, 1678, 1532, 1347. ^1H NMR (DMSO-d_6 from TMS) δ (ppm) for *cis*- (3h′) and *trans*-isomer (3h): 14.06 (s, 1H), 11.95 (s, 1H), 11.31 (s, 1H), 10.77 (s, 1H), 8.76 (s, 1H), 8.65 (s, 1H), 8.50 (m, 2H), 7.13, 8.40 (d, *J* = 7.60 Hz, 1H), 8.30 (d, *J* = 7.88 Hz, 1H), 7.90 (m, 3H), 7.44 (s, 1H), 7.24 (m, 2H), 6.86 (d, *J* = 7.92 Hz, 1H), 6.83 (d, *J* = 7.92 Hz, 1H), 2.31 (s, 6H). ^{13}C NMR δ (ppm) for *cis*- (3h′) and *trans*-isomer (3h): 165.0, 163.5, 148.5, 148.1, 142.4, 140.9,

135.5, 134.8, 134.1, 134.0, 133.0, 132.4, 131.4, 131.1, 130.7, 127.9, 127.6, 127.1, 123.9, 121.9, 120.1, 116.0, 111., 111.0, 21.1, 21.00. $C_{16}H_{12}N_4O_4$ Calcd. C, 59.26, H, 3.73, N,17.28. Found C, 59.35, H, 3.82, N, 17.09.

N'-[5-methyl-2-oxo-1,2-dihydro-3*H*-indol-3-ylidene]-4-(trifluoromethyl)benzohydrazide (4b) Light orange solid, yield 87%, d.t. 313.6 °C, IR (ATR), cm^{-1}: 3247, 1728, 1662, 1483, 1315. ^{1}H NMR (DMSO-d$_6$ from TMS) δ (ppm) for *cis*-(4b′) and *trans*-isomer (4b): 14.00 (s, 1H), 11.80 (s, 1H), 11.31 (s, 1H), 10.75 (s, 1H), 8.14 (d, *J* = 7.88 Hz, 2H), 8.09 (d, *J* = 8.24 Hz, 2H), 8.01 (d, *J* = 8.28 Hz, 2H), 7.95 (d, *J* = 8.24 Hz, 2H), 7.83 (s, 1H), 7.45 (s, 1H), 7.23 (t, 2H), 6.86 (d, *J* = 7.92 Hz, 1H), 6.82 (d, *J* = 7.96 Hz, 1H), 2.32 (s, 6H). ^{13}C NMR δ (ppm) for *cis*- (4b′) and *trans*-isomer (4b): 165.1, 163.5, 142.4, 140.9, 137.4, 136.4, 133.9, 133.0, 132.7, 132.6, 132.4, 132.4, 132.1, 131.8, 131.2, 130.00, 128.9, 128.4, 127.8, 126.6, 125.9, 125.9, 125.7, 125.6, 123.0, 122.8, 121.9, 120.3, 120.1, 116.0, 111.5, 110.9, 21.1, 20.9. $C_{17}H_{12}F_3N_3O_2$, Calcd. C, 58.79, H, 3.48, N, 12.10. Found C, 58.50, H, 3.57, 12.30.

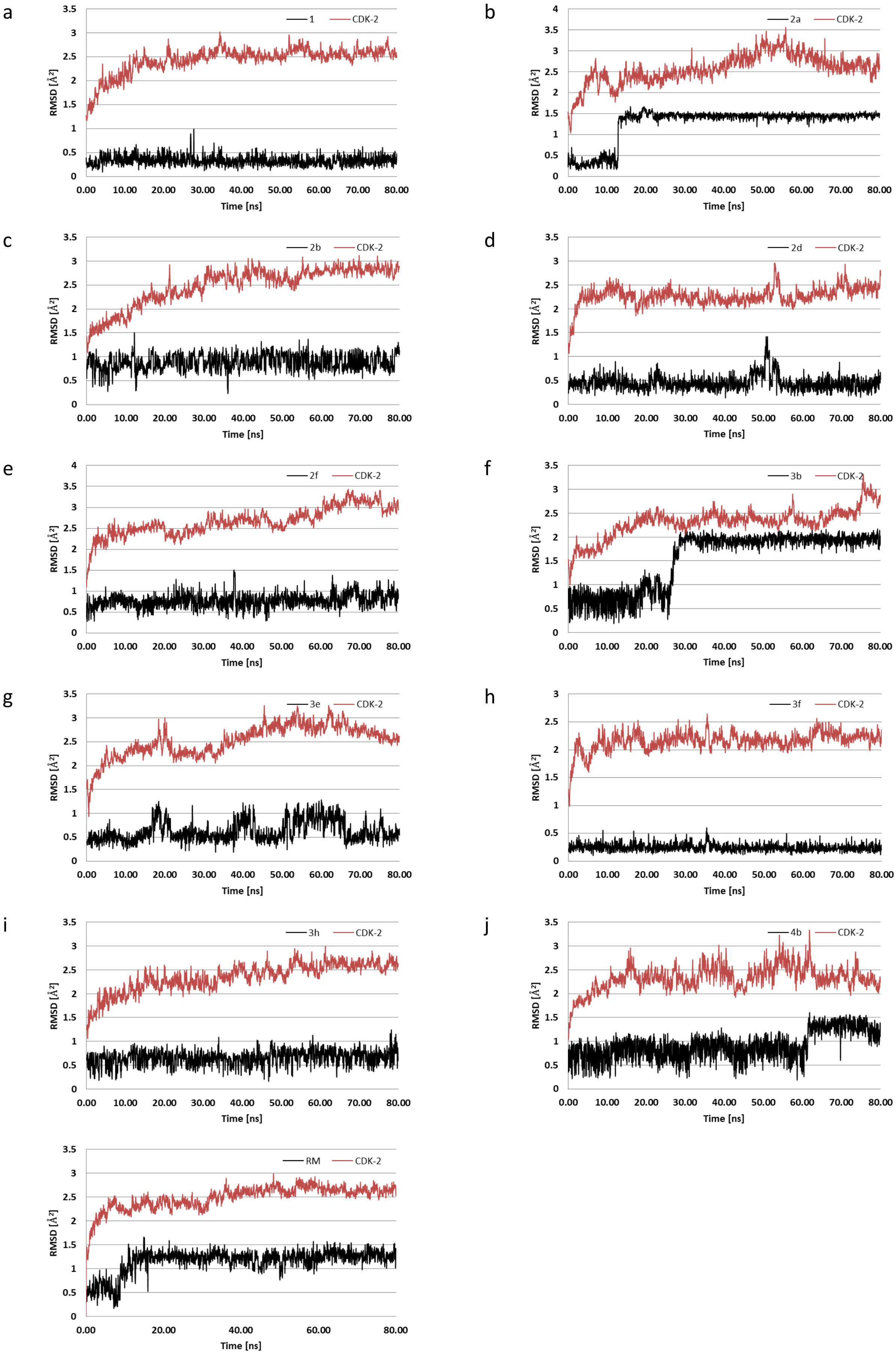

Figure S1. Distributions of RMSD values. Black distributions refer to ligand molecules while red distributions refer to CDK-2 protein.

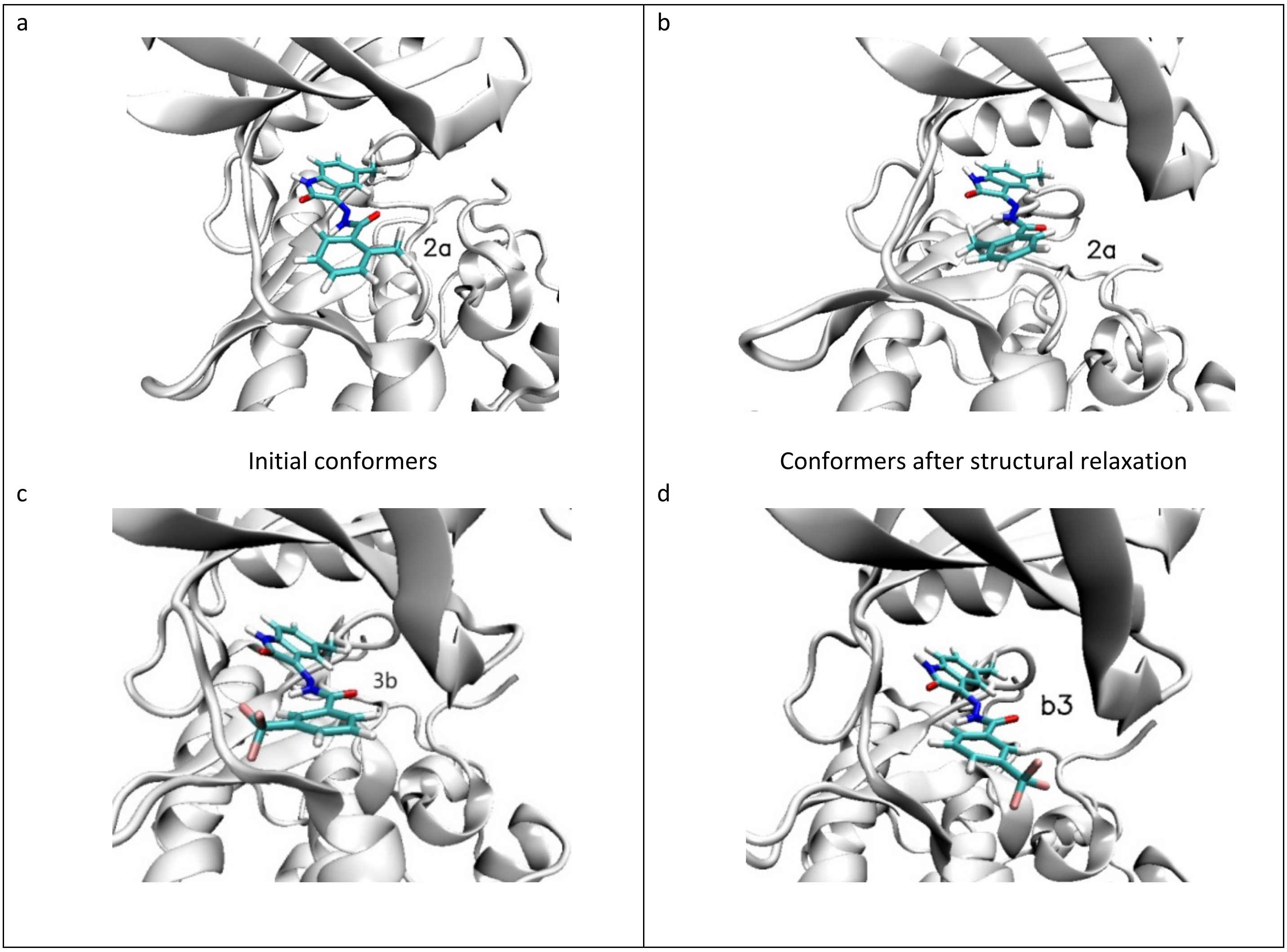

Figure S2. The graphic representation of complexes illustrating conformational changes observed for derivative 2a (a, b) and derivative 3b (c, d).

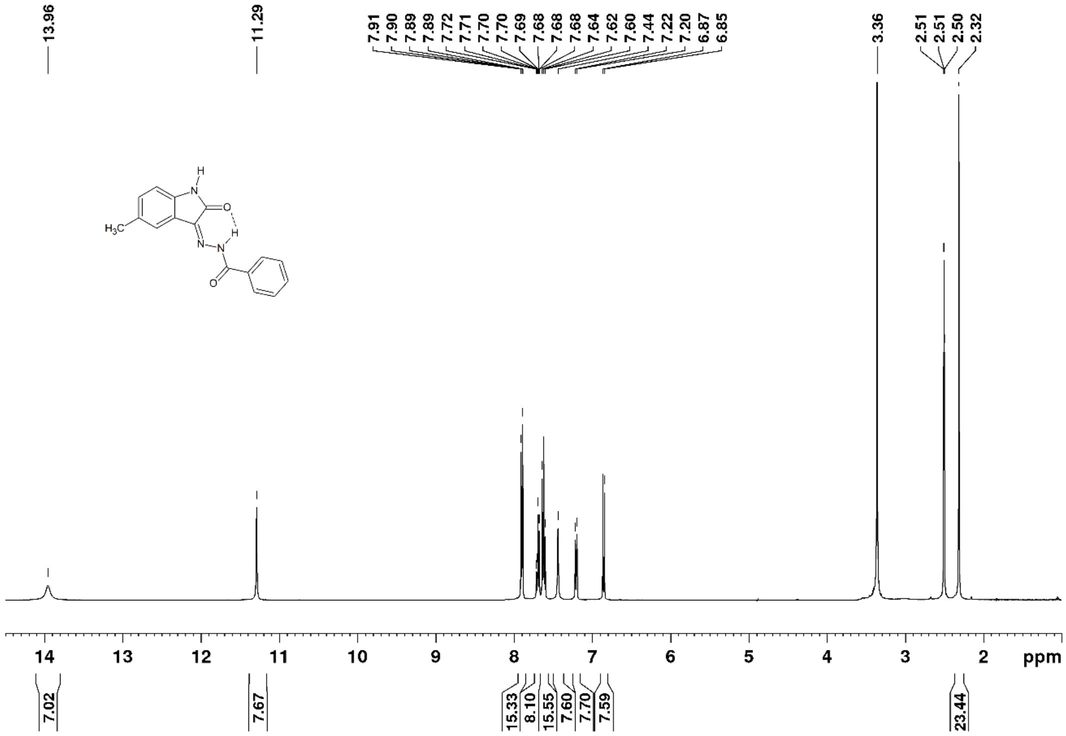

Figure S3. 1H NMR spectrum (400 MHz) of 1 in DMSO-d6.

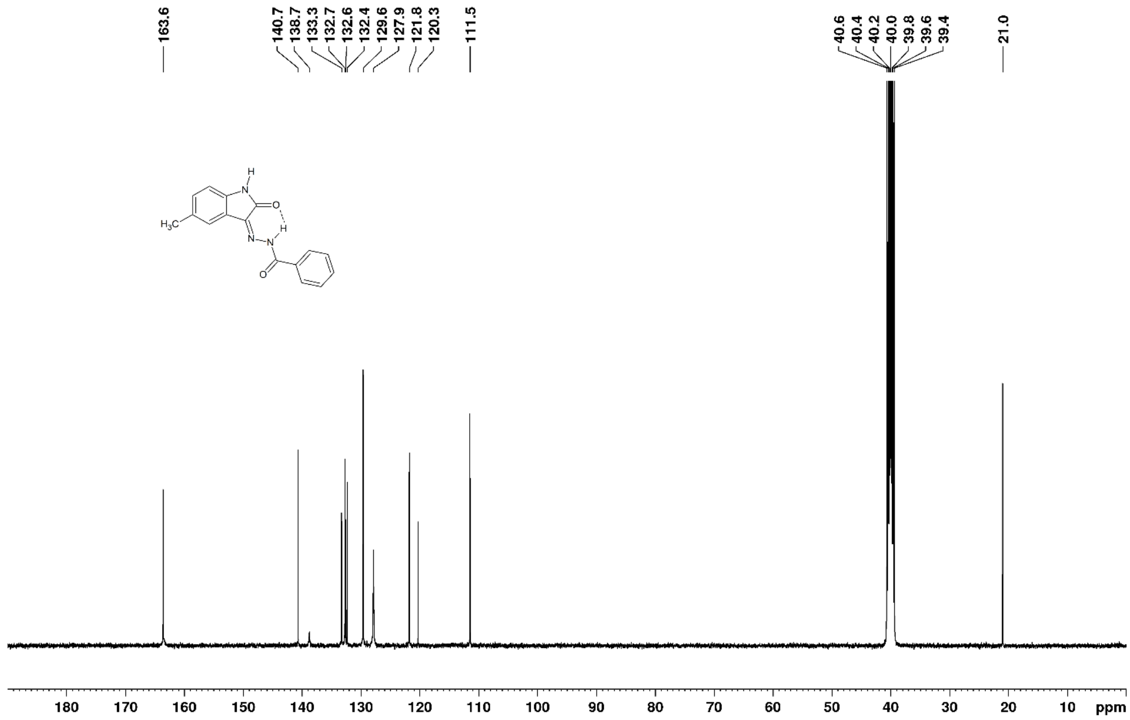

Figure S4. 13C NMR spectrum (100 MHz) of 1 in DMSO-d6.

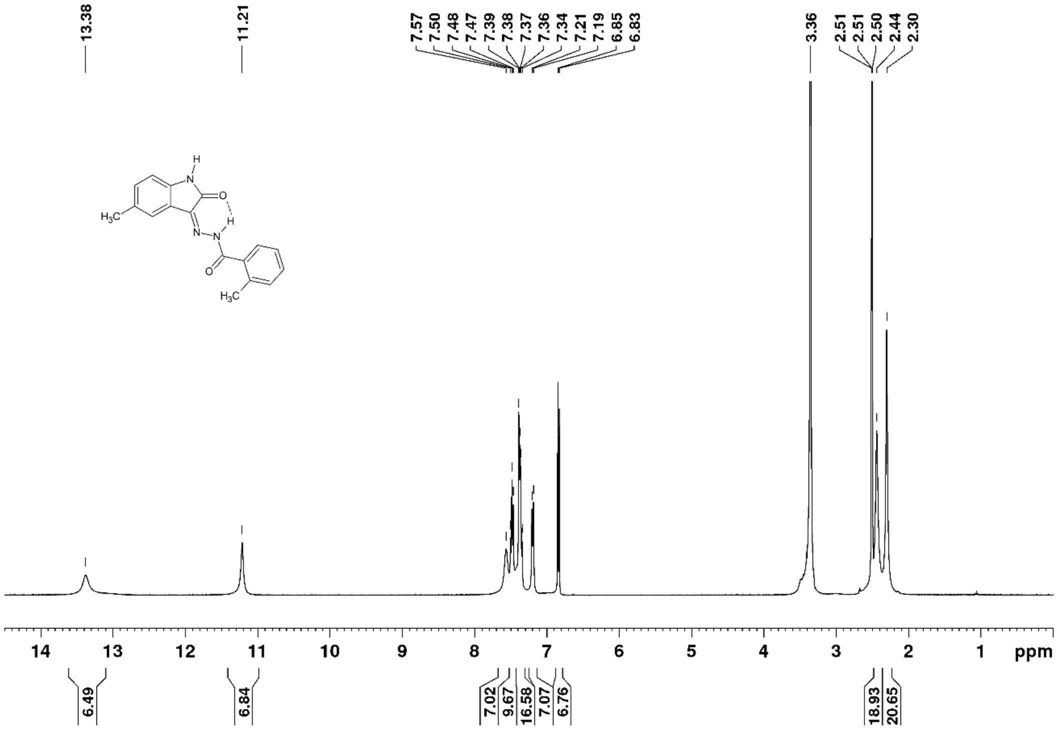

Figure S5. 1H NMR spectrum (400 MHz) of 2a in DMSO-d6.

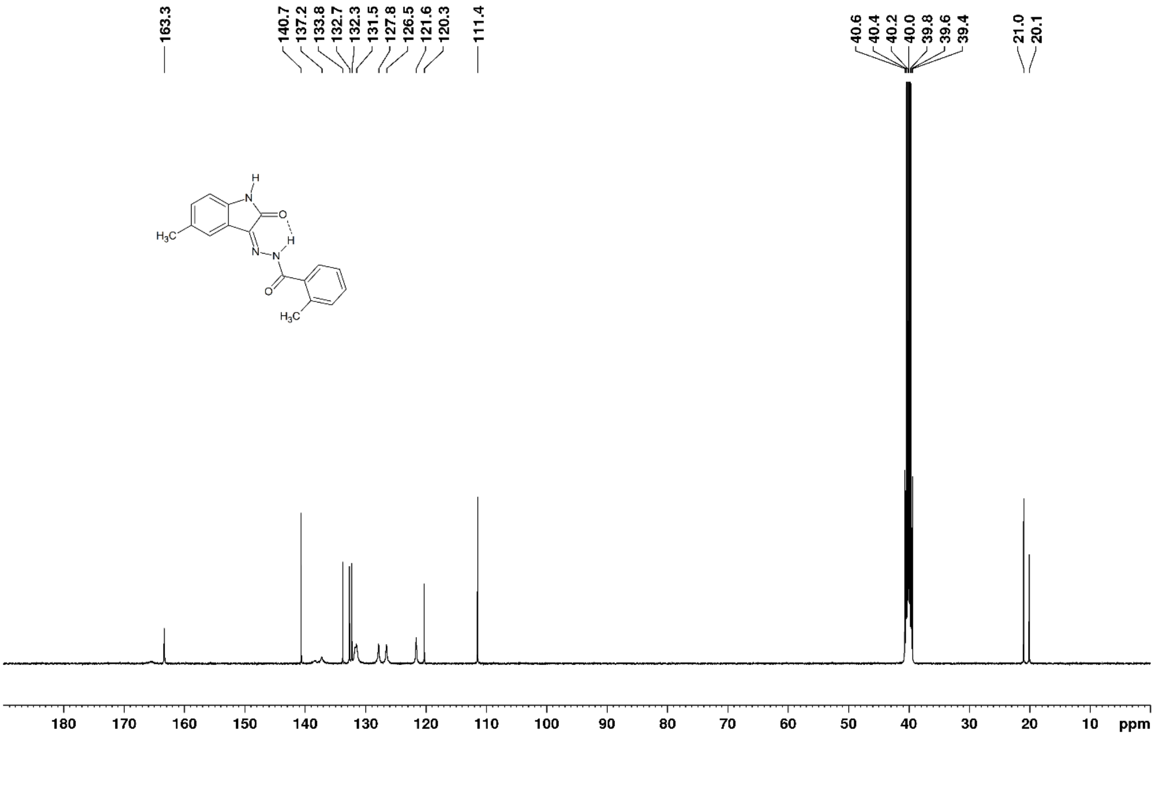

Figure S6. ^{13}C NMR spectrum (100 MHz) of 2a in DMSO-d_6.

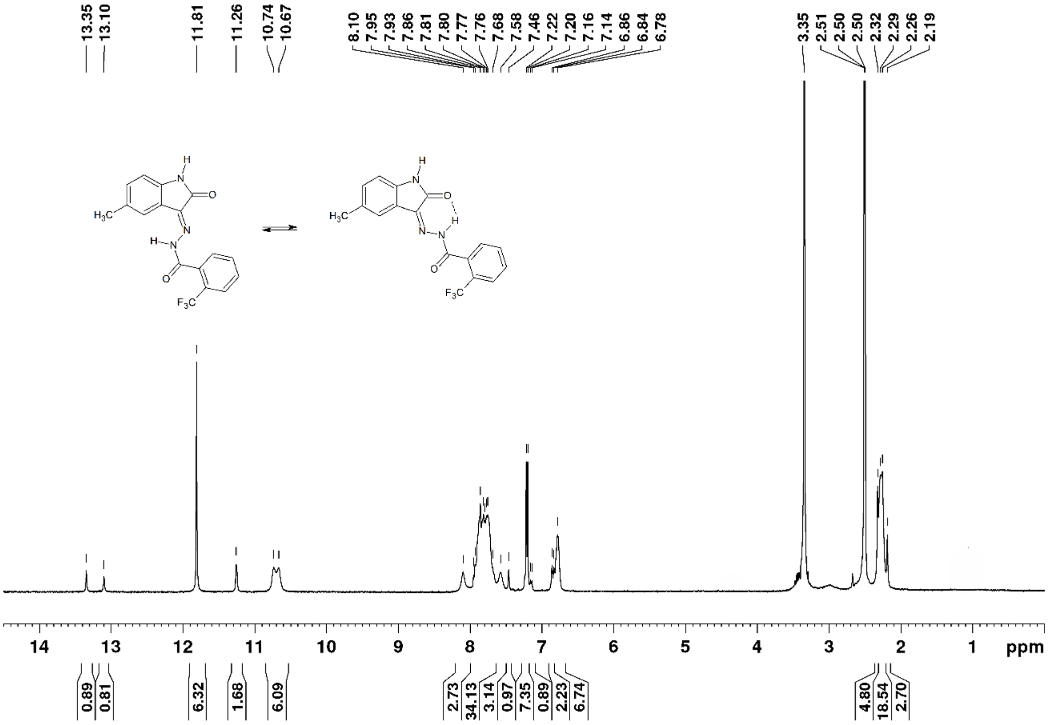

Figure S7. ^{1}H NMR spectrum (400 MHz) of 2b in DMSO-d_6.

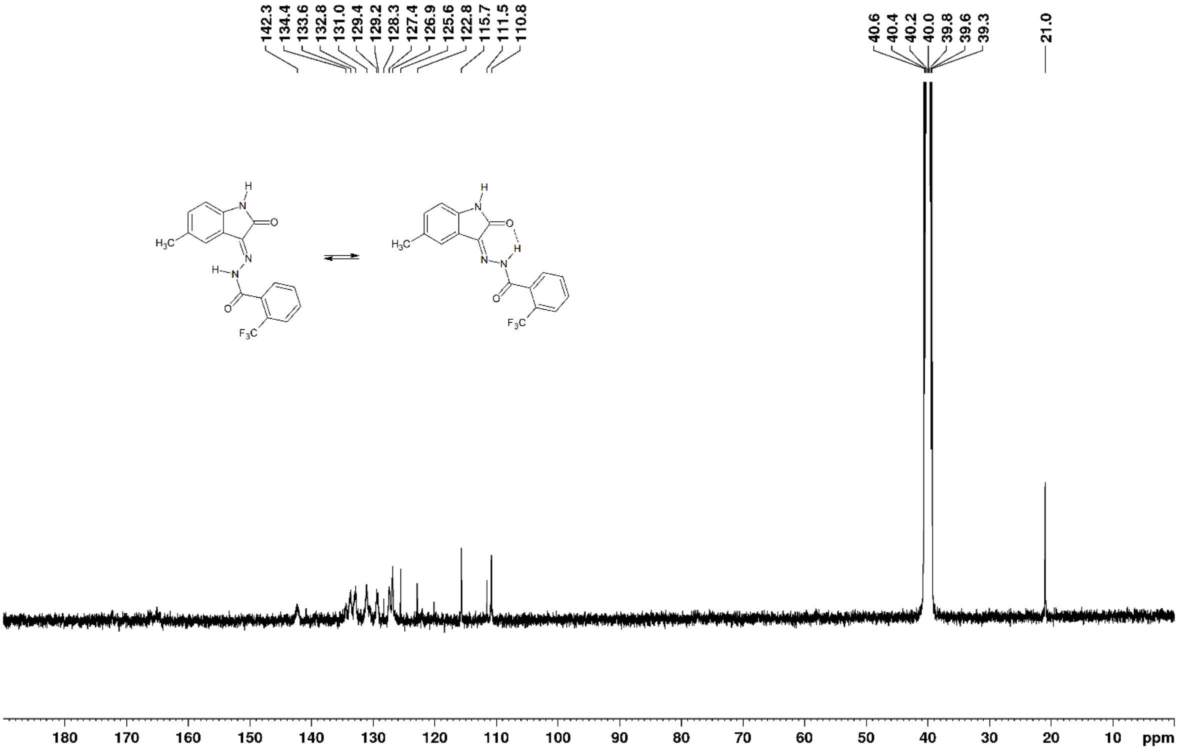

Figure S8. ^{13}C NMR spectrum (100 MHz) of 2b in DMSO-d_6.

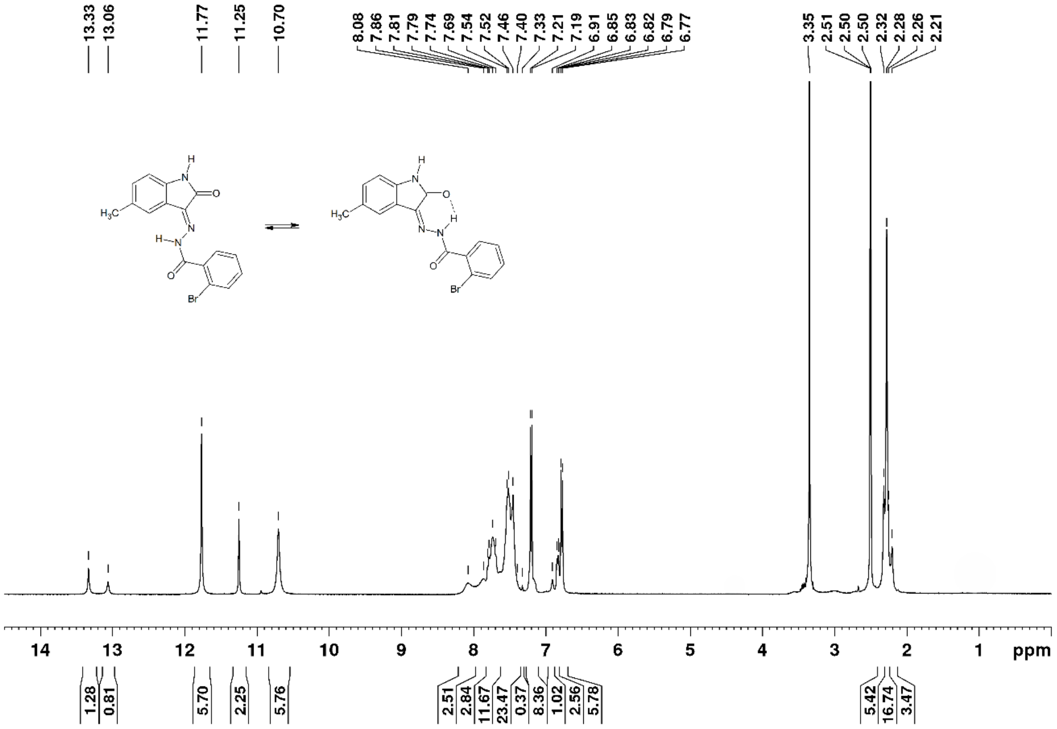

Figure S9. ^{1}H NMR spectrum (400 MHz) of 2d in DMSO-d_6.

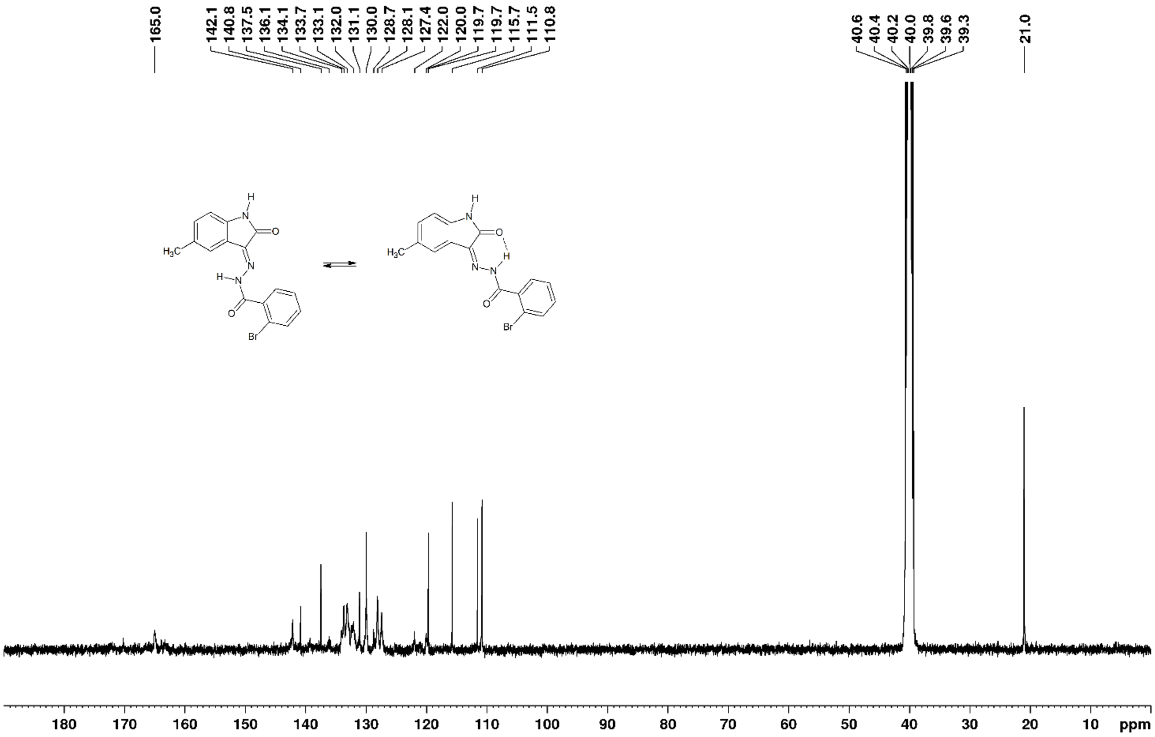

Figure S10. 13C NMR spectrum (100 MHz) of 2d in DMSO-d6.

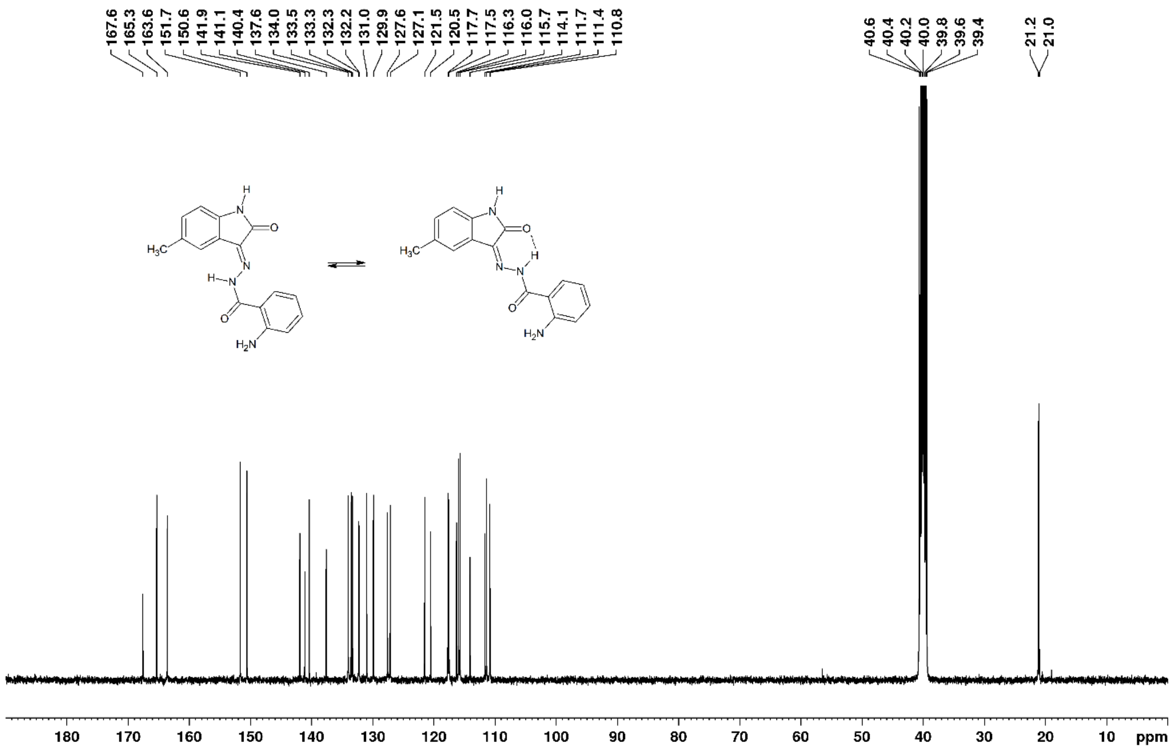

Figure S11. 1H NMR spectrum (400 MHz) of 2f in DMSO-d6.

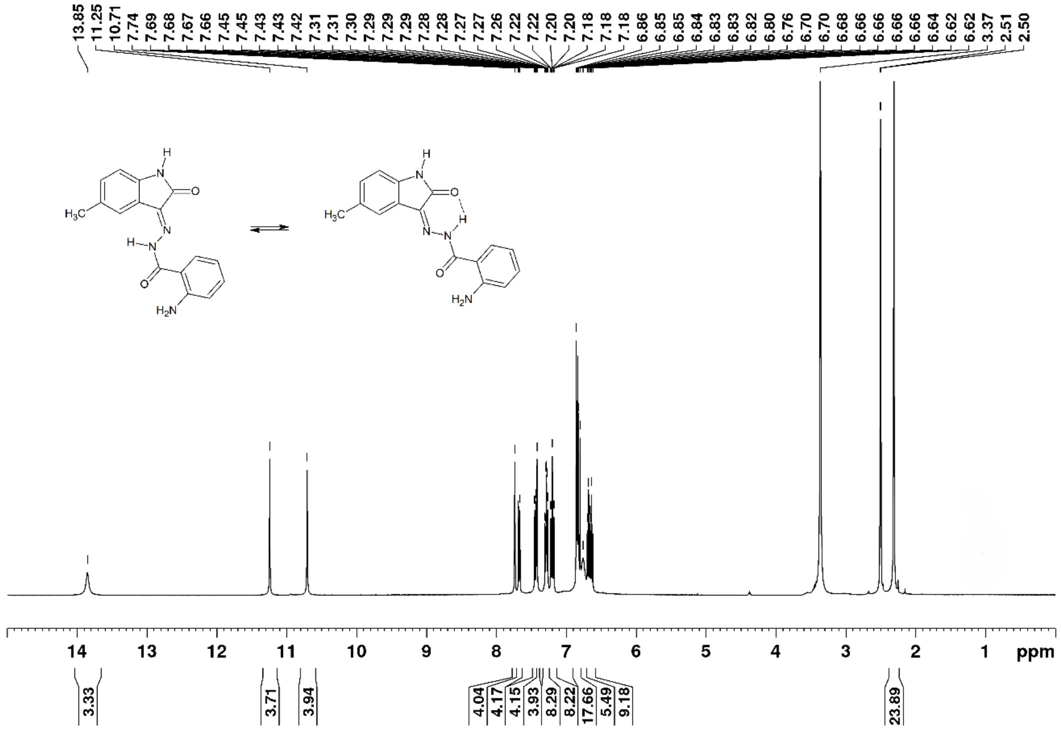

Figure S12. 13C NMR spectrum (100 MHz) of 2f in DMSO-d6.

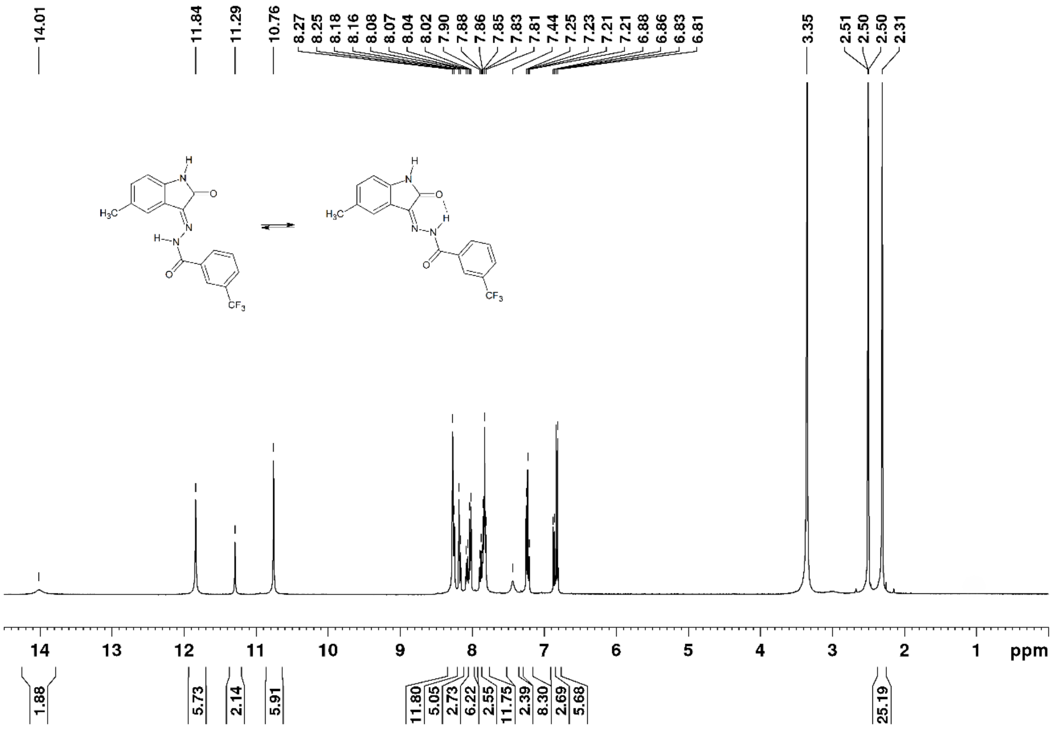

Figure S13. 1H NMR spectrum (400 MHz) of 3b in DMSO-d6.

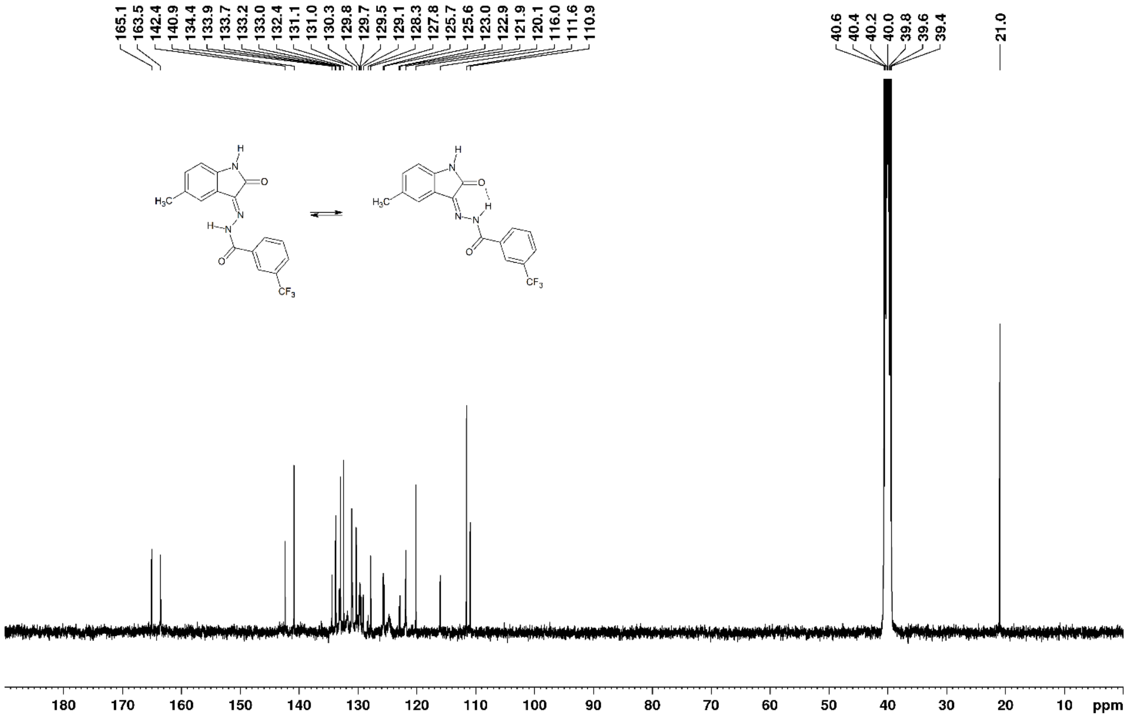

Figure S14. 13C NMR spectrum (100 MHz) of 3b in DMSO-d6.

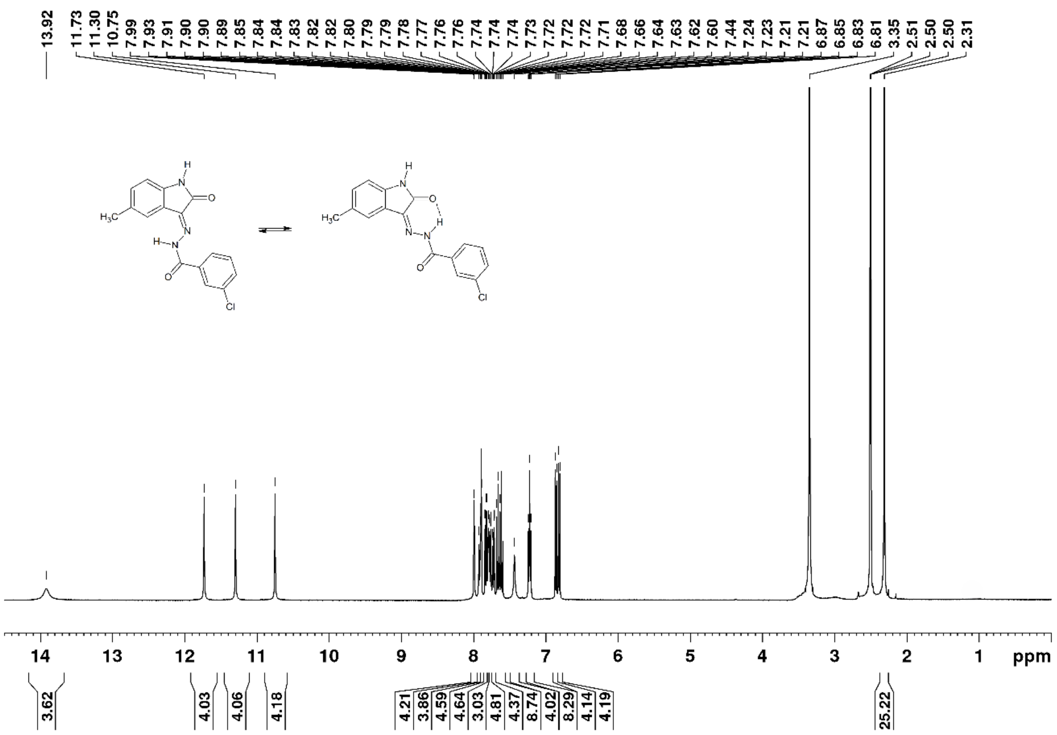

Figure S15. 1 H NMR spectrum (400 MHz) of 3e in DMSO-d6.

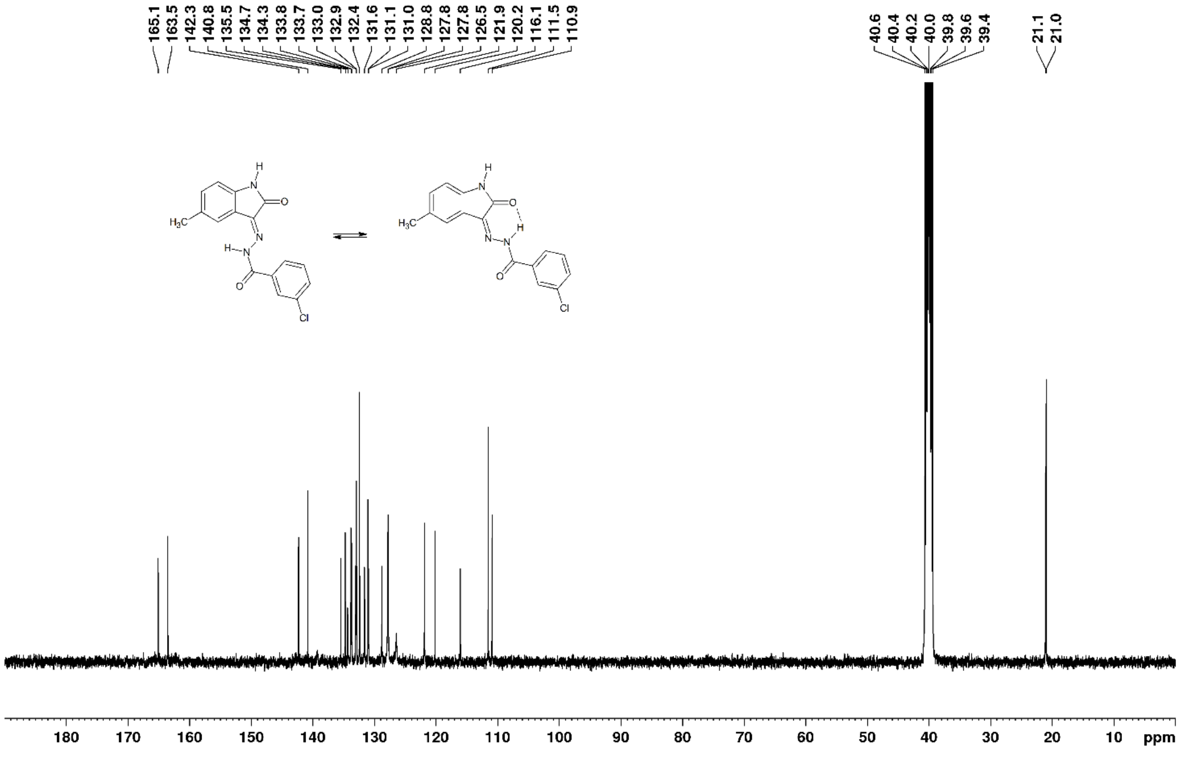

Figure S16. 13C NMR spectrum (100 MHz) of 3e in DMSO-d6.

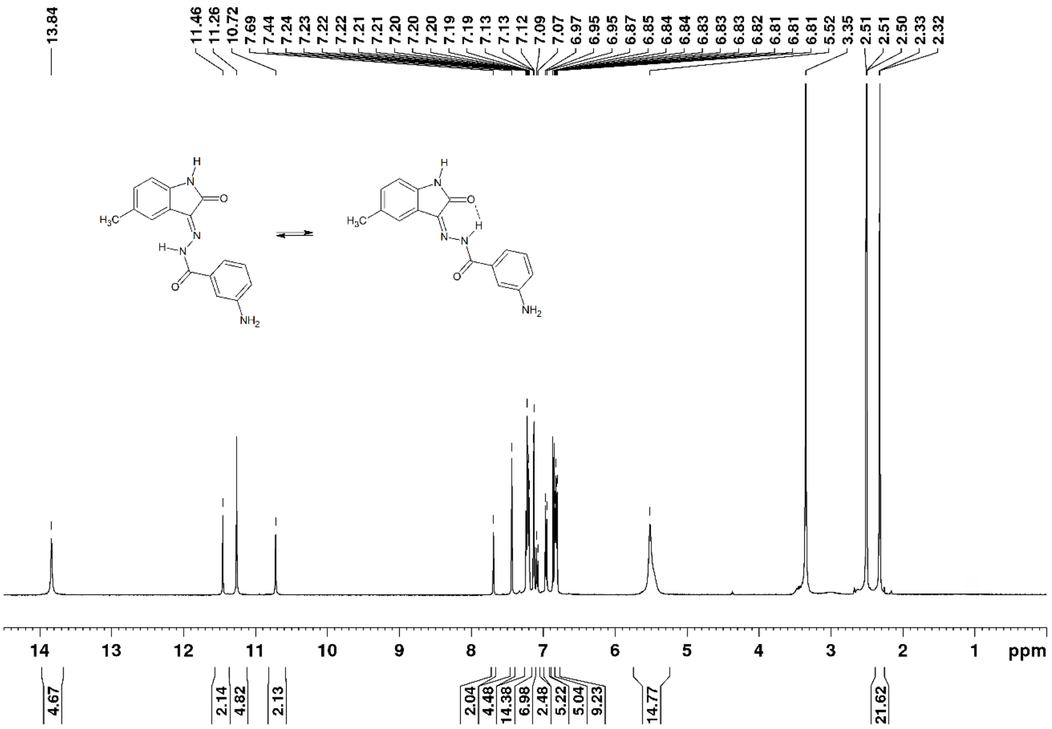

Figure S17. 1H NMR spectrum (400 MHz) of 3f in DMSO-d6.

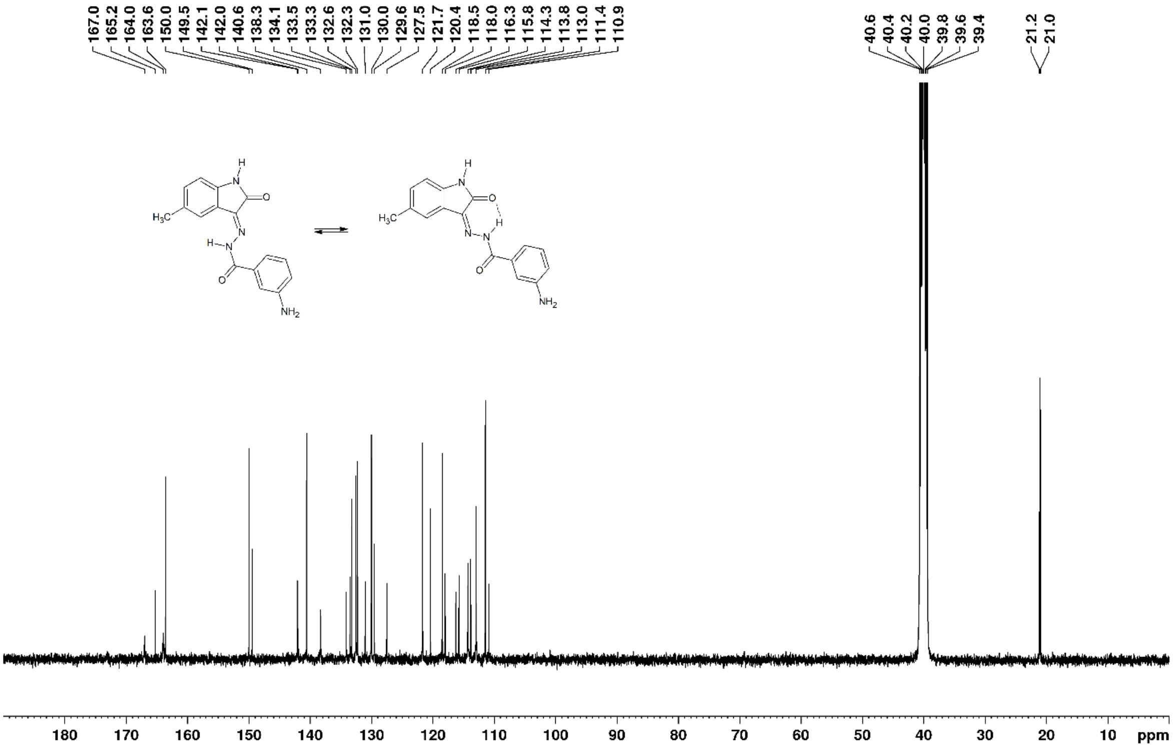

Figure S18. 13C NMR spectrum (100 MHz) of 3f in DMSO-d6.

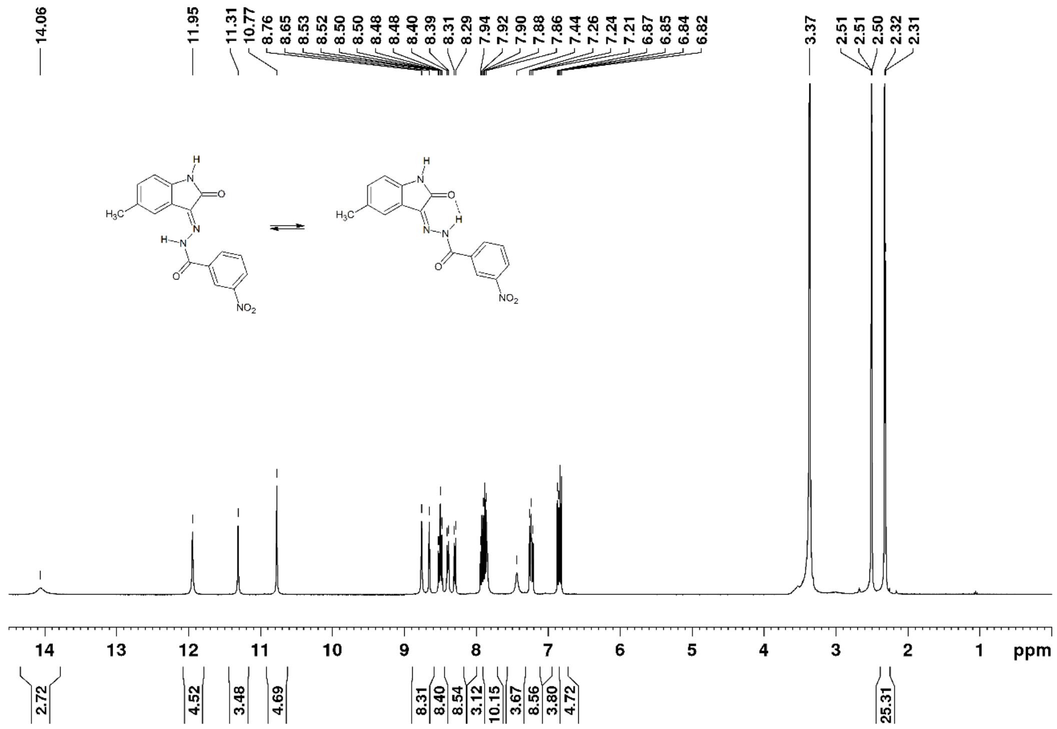

Figure S19. 1H NMR spectrum (100 MHz) of 3f in DMSO-d6.

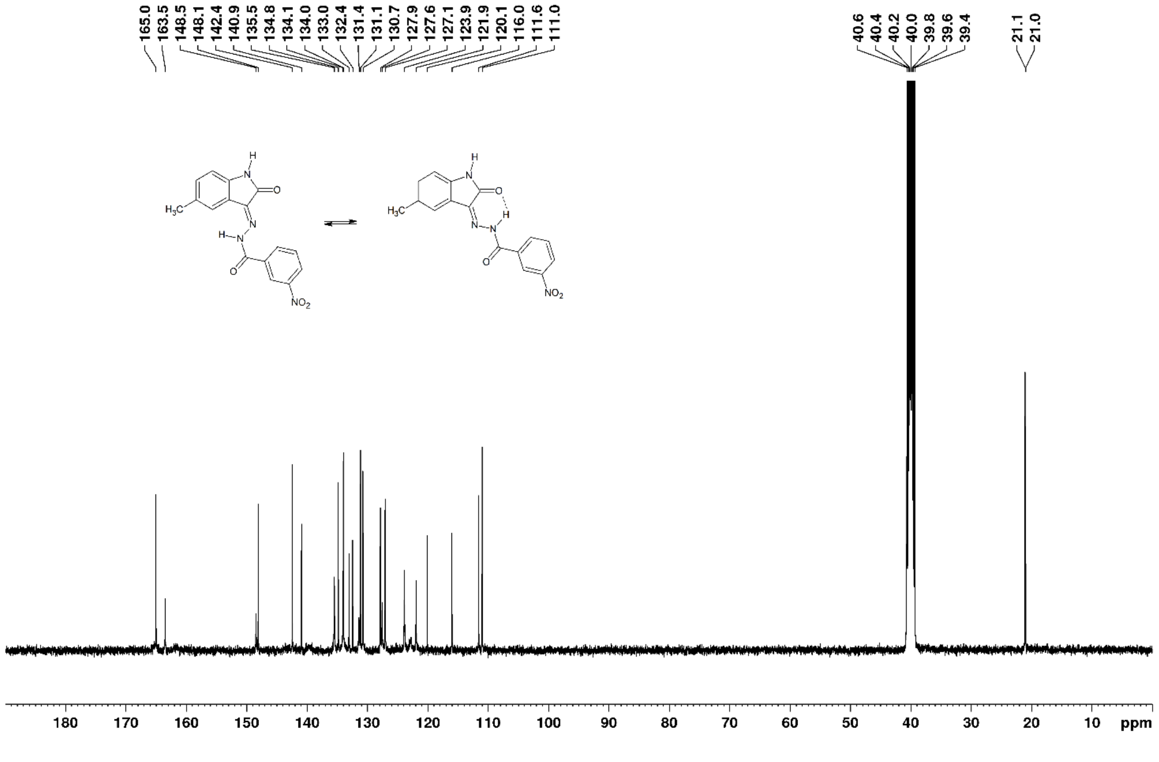

Figure S20. 13C NMR spectrum (100 MHz) of 3f in DMSO-d6.

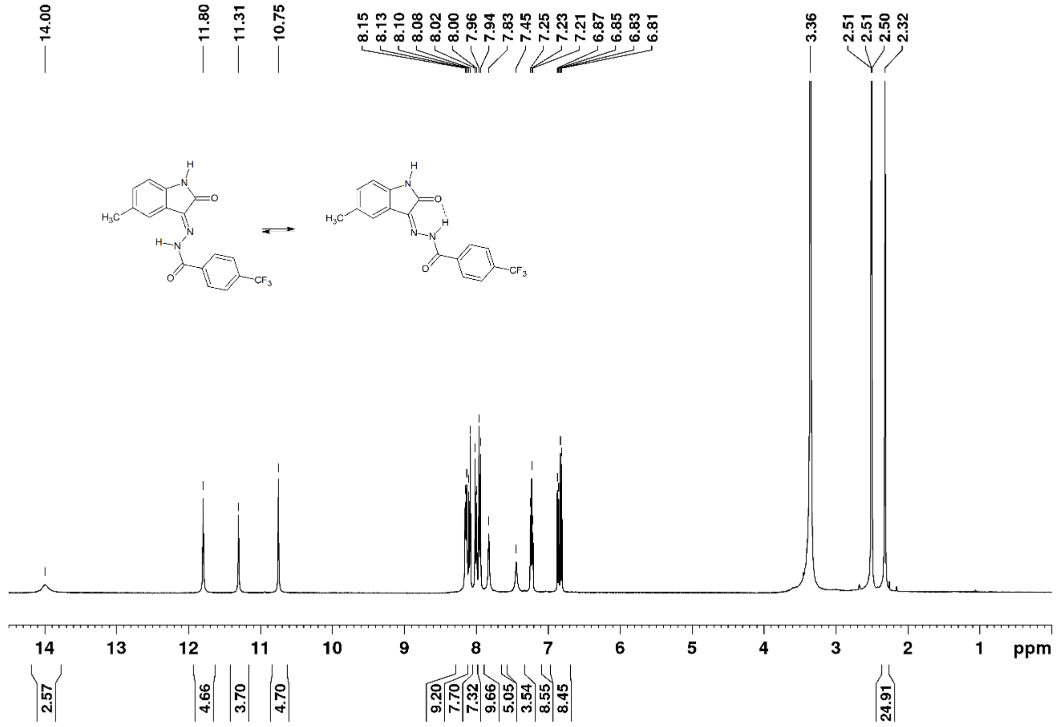

Figure S21. 1H NMR spectrum (400 MHz) of 4b in DMSO-d6.

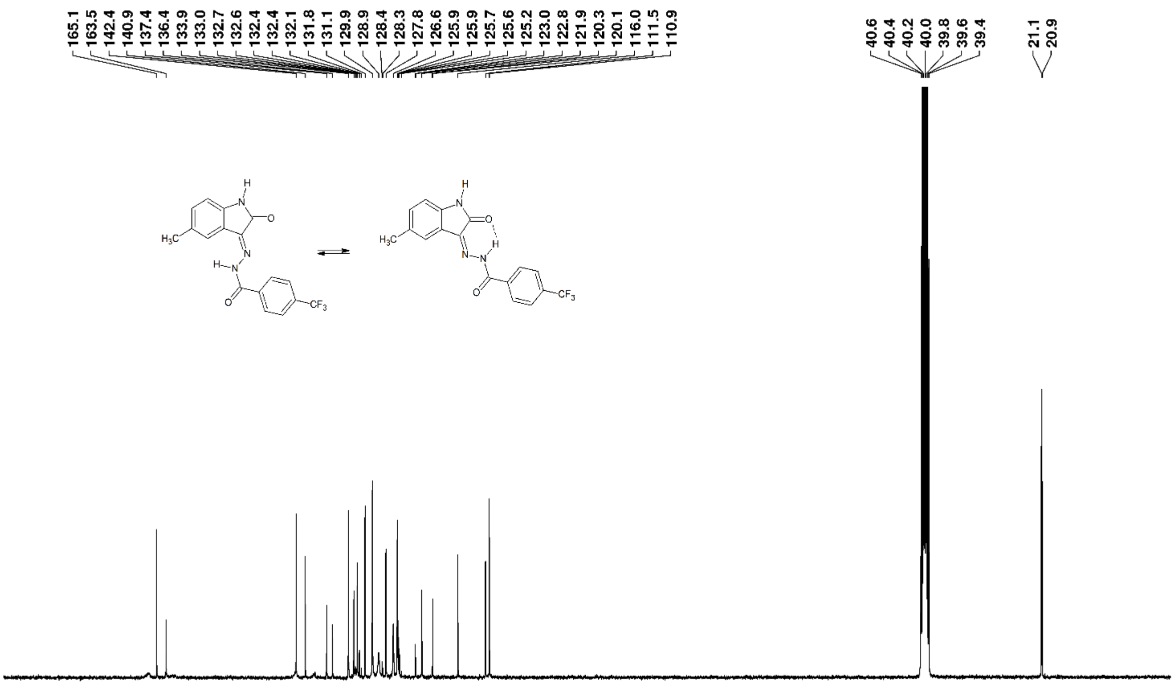

Figure S22. 13C NMR spectrum (100 MHz) of 4b in DMSO-d6.
